# Combined Stress Conditions in Melon Induce Non-additive Effects in the Core miRNA Regulatory Network

**DOI:** 10.3389/fpls.2021.769093

**Published:** 2021-11-25

**Authors:** Pascual Villalba-Bermell, Joan Marquez-Molins, María-Carmen Marques, Andrea G. Hernandez-Azurdia, Julia Corell-Sierra, Belén Picó, Antonio J. Monforte, Santiago F. Elena, Gustavo G. Gomez

**Affiliations:** ^1^Instituto de Biología Integrativa de Sistemas (I^2^SysBio), Consejo Superior de Investigaciones Científicas (CSIC), Universitat de València (UV), Valencia, Spain; ^2^Instituto de Conservacióny Mejora de la Agrodiversidad Valenciana (COMAV), Universitat Politècnica de València (UPV), Valencia, Spain; ^3^Instituto de Biología Molecular y Celular de Plantas (IBMCP), Consejo Superior de Investigaciones Científicas (CSIC), Universitat Politècnica de València (UPV), Valencia, Spain; ^4^The Santa Fe Institute, Santa Fe, NM, United States

**Keywords:** crop production and climate change, miRNAs and stress response in *Cucumis melo*, RNA regulatory networks, RNA-seq and systems biology, biotic and abiotic stress

## Abstract

Climate change has been associated with a higher incidence of combined adverse environmental conditions that can promote a significant decrease in crop productivity. However, knowledge on how a combination of stresses might affect plant development is still scarce. MicroRNAs (miRNAs) have been proposed as potential targets for improving crop productivity. Here, we have combined deep-sequencing, computational characterization of responsive miRNAs and validation of their regulatory role in a comprehensive analysis of response of melon to several combinations of four stresses (cold, salinity, short day, and infection with a fungus). Twenty-two miRNA families responding to double and/or triple stresses were identified. The regulatory role of the differentially expressed miRNAs was validated by quantitative measurements of the expression of the corresponding target genes. A high proportion (ca. 60%) of these families (mainly highly conserved miRNAs targeting transcription factors) showed a non-additive response to multiple stresses in comparison with that observed under each one of the stresses individually. Among those miRNAs showing non-additive response to stress combinations, most interactions were negative, suggesting the existence of functional convergence in the miRNA-mediated response to combined stresses. Taken together, our results provide compelling pieces of evidence that the response to combined stresses cannot be easily predicted from the study individual stresses.

## Introduction

During their life cycle, plants are exposed to a wide array of adverse environmental conditions that, in general, limit their normal development and productivity. These complex interactions result in several stress situations that disturb the homeostasis of the cell, negatively affecting plant growth. Consequently, stress-induced damages in productivity are the primary cause of extensive agricultural losses worldwide (Priya et al., 2019). Reduction in crop yield due to environmental variations has increased steadily over the last decades. In addition, several production models project a reduction in the yields of major agricultural crops in the future, mostly due to climatic changes (Rosenzweig et al., 2014).

Climate change, entailing shifts in temperature, precipitation, and atmospheric composition, among other factors, represents a moving target for plant developmental adaptation. In parallel, environmental modifications can favor the development of new plant pest and/or pathogens or increase the incidence levels of already existing ones. As a consequence of this complex environmental scenario, it is expected that combined abiotic and biotic stresses can affect plants at the level of molecular functions, developmental processes, morphological traits, and physiology, resulting in a significant decrease in crop production and quality (Gray and Brady, 2016; Morales-Castilla et al., 2020).

Multiples studies focused on plant responses to individual stresses have been carried out over the last years. However, less attention has been paid to the effect that combinations of adverse environmental conditions might exert on plant development (Bai et al., 2018). In order to improve crop yield and to meet the growing challenges stemming from rapid population growth, extensive efforts are needed to understand the mechanisms underlying plant responses to simultaneous exposure to multiple stresses (Zhang and Sonnewald, 2017). Previous works have pointed out that studying stress conditions separately would not allow inferring the expected plant response to multiple stresses. Using *Arabidopsis thaliana* as an experimental model, it was shown that the response to a combination of drought and heat was unique and could not be directly extrapolated from the plant response to each stress applied individually (Rizhsky et al., 2004; Suzuki et al., 2005; Rossel et al., 2007). Similar findings were also reported for a combination of heat and high light intensity in sunflower (Hewezi et al., 2008), and heat and salinity in wheat (Keleş and Öncel, 2002). Consequently, plant response to combined adverse environmental conditions should be handled as a new state of stress that requires a novel conceptual viewpoint (Mittler and Blumwald, 2010).

In general, plants respond to stress conditions through complex reprogramming of their transcriptional activities, aiming to reduce the impact of stress on their physiological and cell homeostasis. Environmental variations have selected diverse responses among plant lineages, landraces, and wild crops relatives. Studies on natural variations can provide novel insights into evolutionary processes modulating stress response (Meyers et al., 2008; Haak et al., 2017). Elucidation of how endogenous regulators and the environment interact during plant development is a long-standing grand challenge in modern biology as well as in crop breeding (Lovell et al., 2015).

MicroRNAs play a versatile role as regulators of gene expression. Plant genes-encoding miRNAs are transcribed by RNA polymerase II as primary transcripts harboring a fold back structure that is processed by DICER-LIKE 1 (DCL1) in a duplex (21 or 22 nt in length), which once 2′-*O*-methylated by HEN1 is loaded into an AGO complex (Bartel, 2004; Bologna and Voinnet, 2014; Reis et al., 2015; Achkar et al., 2016). miRNAs regulate gene expression by means of sequences complementarity with both RNA and DNA targets (Song et al., 2019). Their functions include modulation of a vast array of plant biological processes related to grown and development (Bologna and Voinnet, 2014), including the recovering of the plant-cell homeostasis during exposure to adverse environmental condition (Song et al., 2019; Xu et al., 2019). In addition, it has been recently described that the biogenesis and turnover of certain miRNAs are also susceptible to be controlled by external stimulus (Bustamante et al., 2018; Manavella et al., 2019). Indeed, it has been proposed that miRNAs are ideal targets to be manipulated to improve crop productivity (Tang and Chu, 2017; Xu et al., 2019). However, most of the described stress-responsive miRNAs come from rice and tomato, as very few miRNAs have been investigated in detail in other crops. Henceforth, additional efforts are needed to decipher the role of miRNA-mediated responses to adverse environmental conditions in other economically relevant crops (Tang and Chu, 2017).

Although increasing pieces of evidence support the role of miRNAs as key modulators of plant response to both biotic (Sun et al., 2017; Xie et al., 2017; Brant and Budak, 2018) and abiotic stress conditions (Cervera-Seco et al., 2019; Wang et al., 2020; Cheng et al., 2021; Zhao et al., 2021), research focusing on elucidating the regulatory role of the miRNAs during exposure to combined adverse environmental conditions is still scarce (Xu et al., 2019), and only a few studies considering the effects of a unique combination of stresses have been addressed in soybean (Ning et al., 2019), *A. thaliana* (Gupta et al., 2020), wheat (Liu et al., 2020), and tomato (Zhou et al., 2020).

Melon (*Cucumis melo*) is one of the cucurbit crops with more economic impact. Melon has high adaptability to warm and dry climates, so it can be a target crop to cope with the climate change threats. Previous genetic studies in cucurbits have been focused mainly on fruit quality and disease resistance (Gonzalo and Monforte, 2017). However, the study of the response to combined stress conditions has not been thoroughly addressed in cucurbits. Consequently, there is a lack of consensus protocols, target traits, and, therefore, identification of tolerant genotypes to develop efficiently resilient cultivars.

Here, we used deep-sequencing, computational approaches and specific miRNA-targets quantification to present a comprehensive functional analysis of miRNA expression profiles in response to one triple (cold, salinity, and short day) and five double (cold and drought, cold and salinity, cold and short day, drought and salinity, and drought and infection with the fungus *Monosporascus cannonballus*) combinations of stress conditions in melon (*Cucumis melo*), a crop extensively cultivated in semiarid regions worldwide. The analyzed stress conditions were coincident, in part, with those employed recently to infer the miRNA-mediated regulatory network of response to individual stresses in melon (Sanz-Carbonell et al., 2019, 2020). The parallelism between both simultaneous experimental approaches made possible to unambiguously analyze the effects that the combined adverse environmental conditions have on the accumulation of the stress-responsive miRNAs.

## Materials and Methods

### Plant Material, Growth Conditions, and Stress Treatments

Melon seeds of cv. Piel de Sapo were germinated in Petri dishes at 37°C/48 h in darkness, followed by 24 h/25°C (16/8 light/darkness). Melon seedlings were sown in pots and maintained for 10 days under controlled conditions (28°C/16-h light and 20°C/8-h darkness). At day 11, plants were exposed to six stress-combined treatments (detailed in Supplementary Table 1). We selected abiotic conditions well established as crucial for plant development (cold, salinity, and short day) and *Monosporascus cannonballus* (a soil-borne fungal pathogen capable of causing root rot and wilting in melon (Pollack and Uecker, 1974) as biotic inducers of stress. At 11 days post-treatment, the first leaf under the apical end per plant was collected in liquid nitrogen and maintained at –80°C until processing. Each analyzed sample corresponds to a pool of three treated plants. Three biological replicates were performed per treatment. Leaves recovered from non-treated plants were considered as controls. This stress assay was performed simultaneously with the recent work describing the response to single stress conditions in melon (Sanz-Carbonell et al., 2019).

### RNA Extraction and Small RNA Purification and Sequencing

Total RNA was extracted from leaves (∼0.1 g) recovered from treated and control melon as previously described (Sanz-Carbonell et al., 2019, 2020). The low-molecular weight RNA (<200 nt) fraction was enriched from total RNA using TOTAL-miRNA (miRNA isolation Kit, REAL) according to the manufacturer’s instructions. Production and sequencing of the libraries were carried out by Novogene^1^. Eighteen cDNA libraries were obtained by following Illumina’s recommendations and sequenced in HiSeq 2000 (Illumina) equipment. Adaptors and low-quality reads were trimmed by using the cutadapt software (Martin, 2011). For the sake of comparing the results generated in here with those obtained for single stresses, data previously obtained from melon plants exposed to identical single stress conditions for 11 days (Sanz-Carbonell et al., 2019) were also included in the study. Melon miRNA sequences used in this study have been submitted to the genomic repository SRA of the NCBI and are available in the BioProject (PRJNA741881).

### RT-qPCR Assays

To analyze the expression of target genes and miRNA precursors, total RNA (1.5 μg) was subjected to DNase treatment (EN0525, Thermo Scientific™), followed by reverse transcription using RevertAid First Strand cDNA Synthesis Kit (Thermo Scientific™) according to the manufacturer’s instructions for use with oligo-dT. cDNAs were amplified by conventional end-point RT-PCR using specific primers to assess for sequence specificity. Then, real-time PCR was performed as described previously (Bustamante et al., 2018). All analyses were done in triplicate on a QuantStudio qPCR instrument (Thermo Scientific™) using a standard protocol. The efficiency of PCR amplification was derived from a standard curve generated by four 10-fold serial dilution points of cDNA obtained from a mix of all the samples. Relative RNA expression was quantified by the comparative ΔΔ*C*_*T*_ method (Livak and Schmittgen, 2001) and normalized to the geometric mean of Profilin (NM_001297545.1) expression. The statistical significance of the observed differences was evaluated by the paired *t*-test. Primers used for miRNA-targets amplification and profiling were described previously (Sanz-Carbonell et al., 2019). Primers used to analyze miRNA precursors are detailed in Supplementary Table 9.

### Bioinformatic Analysis of miRNA Sequences

To study the correlation exhibited by the miRNA expression profiles among the different stresses and their biological replicates, principal component analysis (PCA) was used. PCA was performed using the prcomp function with scaling in the stats R package v. 4.0.4 (R Core Team, 2013). Mann–Whitney–Wilcoxon tests were performed to assess for significant differences in the data clusters for Euclidean distances calculated between groups and among groups with the wilcox.test function in the stats R package.

Differential expression of melon small RNAs (sRNAs) was estimated using three R packages NOISeq (Tarazona et al., 2015), DESeq2 (Love et al., 2014) and edgeR (Robinson and Oshlack, 2010) for pairwise differential expression analysis of expression data. Differentially expressed sRNAs were filtered out using three criteria: (i) log_2_-fold change | log_2_*FC*| ≥ 1.25, (ii) adjusted *p* ≤ 0.05 (DESeq2 and edgeR), probability ≥ 0.95 (NOISeq), and (iii) RPMs ≥ 5 for at least three libraries in control samples or at least two libraries in any stress. sRNAs identified as responding to stress by the three methods were aligned against miRNA sequences in miRBase (release 22) (Kozomara et al., 2019). To generate robust knowledge suitable to be transferred to diverse plant species, only sequences fully homologous to previously described mature melon miRNAs and known *Viridiplantae* miRNAs with well-established regulatory roles were kept.

Afterward, these sequences were re-annotated by aligning them against miRNA precursors of melon deposited in miRBase and were considered as known stress-responsive miRNAs. Unaligned sequences were realigned allowing for one mismatch against the melon genome to identify potential precursors. These sequences were also identified as known stress-responsive miRNAs; the rest were discarded. The entire pipeline is shown in Supplementary Figure 1.

To determine the general sense of the expression for each miRNA family, we employed the median value of expression estimated by box-plot analysis of all family-related sequences under each stress condition, considering the log_2_*FC* values obtained by edgeR. The most frequent sequence in each miRNA family and stress was used to generate heatmaps with an R interface to a morpheus.js heatmap widget^2^.

### Analysis of the Stress Combination Effect

The expression of reactive miRNAs in response to combined stress conditions can be enfolded in at least one of the three following categories: (i) additive if the observed response to combined stresses is just the sum of the magnitude responses observed for each individual stress, i.e., this represents the null hypothesis of independent actions, (ii) negative if the observed response is smaller than the expected additive response, and (iii) positive if the observed value is greater than the expected additive response. In this framework, if a given miRNA shows an additive response upon exposure to two stresses, it can be assumed that both stresses trigger independent miRNA-mediated responses. In contrast, a miRNA showing a significantly negative or positive deviation from the null hypothesis shall be taken as indicative of a specific response to the combined stresses beyond the simple additive case. To quantitatively test the null hypothesis of additive effects on miRNA-mediated response to stress combinations, we defined an *stress combination effect* (*SCE*) index that refers to the miRNA response value to combined stresses in comparison to what should be expected from individual stress conditions as *SCE* = (*C* + *S*_*ij*_) − (*S*_*i*_ + *S*_*j*_), where *C* refers to the means of the normalized reads recovered in control, *S*_*ij*_ to the reads observed in plants exposed to combined stresses *i* and *j* and *S*_*i*_ and *S*_*j*_ to the reads arising from each individual stress (Supplementary Tables 6A,B). Under the null hypothesis of purely additive effects of stresses *i* and *j* on miRNA expression, the observed expression under both stresses will be equal to the sum of expression observed for each individual stress and hence *SCE* = 0. Positive (*SCE* > 0) or negative (*SCE* < 0) deviations from this null hypothesis will result from an over- or under-expression of the miRNA when stresses *i* and *j* are combined, respectively. For the triple stress condition (*S*_*ijk*_) and additional value (*S*_*k*_)—referred to the means of normalized reads in the additional stress condition *k*—should be added to the second terms of the equation. As written above, for the action of two combined stresses *i* and *j*, it is straightforward to show mathematically that *SCE* is equivalent to the coefficient of the interaction term between stresses *i* and *j* in a two-way ANOVA model. The statistical significance of the *SCE* was calculated on the basis of a standard normal distribution and the adjusted *p-*value by the false discovery rate (FDR) approach. Only the miRNAs with a *p*-value equal or smaller than its respective FDR *q*-value were considered as reliable indicators of effects of stress combinations onto miRNA accumulation (Supplementary Table 6A). Reads exhibiting zero means values in any of the analyzed combinations were filtered out. The data associated with the miRNA expression under single stress conditions were extracted from a previous work, analyzing the differential expression of melon miRNAs in response to seven biotic and abiotic single stress conditions (Sanz-Carbonell et al., 2019). The statistical significance of *SCE* was calculated on the basis of a standard normal distribution. Then, the 22 stress-responsive miRNA-families were organized in a binary table of presence and absence (Supplementary Table 8), in which the values one and zero represent, respectively, whether or not a miRNA family has at least a member exhibiting a significant non-additive (positive or negative) effect in response to a combined stress condition. The hclust function in stats R package (v. 4.0.4) was used to compute hierarchical clustering (HC), specifying Ward linkage (ward.D) as an agglomeration method and using the simple matching coefficient metric to calculate the distance matrix. The statistical significance of the HC was estimated with a Mann–Whitney–Wilcoxon test.

## Results

### Stress Combinations and sRNAs Dataset

High-throughput sequencing of sRNAs was performed, starting from 22 (three replicates for each stress condition plus four non-treated controls) sRNA libraries constructed with RNA extracted from leaves of melon plants 11 days after exposure to six (five double and one triple) combined stress conditions: (i) cold and drought (C-D), (ii) cold and salinity (C-Sal), (iii) cold and short day (C-SD), (iv) drought and salinity (D-Sal), (v) drought and *M. cannonballus* infection (D-Mon), and (vi) cold, salinity, and short day (C-Sal-SD) (Supplementary Table 1). As has been pointed in section “Materials and Methods,” this assay was performed in parallel and simultaneously with our previous work, analyzing the response to single stressors in melon (Sanz-Carbonell et al., 2019). Regarding the stress conditions analyzed, we selected abiotic conditions well established as crucial for melon plant development (cold, drought, salinity, and short day) and infection with *M. cannonballus*, a soil-borne fungal pathogen, causing root rot and wilting in melon (Pollack and Uecker, 1974). Only sequences with size ranging between 20 and 25 nt in length and non-matching to rRNA, tRNA, snoRNA, and snRNA sequences deposited in the Rfam data base^3^ were further included in this study. A total of 80,620,994 reads (representing 36,836,230 unique sequences) were recovered. The distribution of reads by stress condition is detailed in Supplementary Table 2.

**FIGURE 1:**
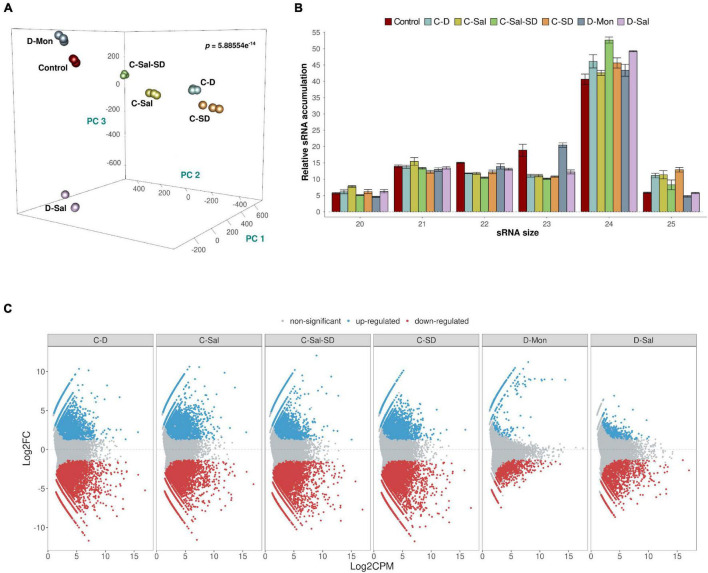
Analysis of the sRNA populations. **(A)** PCA based on sRNAs accumulation in three biological replicates of melon plants exposed to the six stress-combined treatments and controls. The statistical significance (*p* = 5.886 × 10^– 14^) was estimated by Mann–Whitney–Wilcoxon test, considering the inter- and intragroup Euclidean distances. **(B)** A diagram showing the relative accumulation (and distribution of the total clean reads of melon sRNAs ranging between 20 and 25 nt obtained from the analyzed sequenced libraries. The control and the different analyzed treatments are represented with colors. The shown values represent the sum of all repetitions. Bars indicate the standard error. **(C)** Graphic representation of the expression values (estimated by edgeR) of sRNA sequences recovered from melon exposed to different stress conditions. The dots indicate the expression value of each sRNA. Red and blue dots indicate significant values for differential expression with | log_2_*FC*| ≥ 1.25, respectively. Gray dots indicate sRNAs with non-significant differential expression.

Associations between sRNA expression profiles (considering the different treatments and their biological replicates) were evaluated using PCA. The percentages of variance explained by the first three PCs were 20.4, 17.1, and 13.8%, respectively (adding up to 51.3% of the total observed variance). The PCA plot in Figure 1A shows that biological replicates clustered together (attesting for the reproducibility of our assays) and treatments clearly separated in the PC space with high significance (*p* = 5.886 × 10^–15^). The sRNAs exhibited a distribution of read lengths strongly enriched for 24-nt long (45.7%), followed by similar accumulations of 21- (13.5%), 22- (12.6%), and 23 (13.5%)-nt long molecules. As expected, reads of 20 and 25 nt represented the less-abundant categories (5.9 and 8.5%, respectively) (Figure 1B). These differences in accumulation of different sRNA lengths were statistically significant (two-way non-parametric ANOVA, Supplementary Table 3A *p* < 10^–5^). The effect was entirely due to the large enrichment in 24-nt-long sRNAs (Dunn’s *post hoc* pairwise tests, Supplementary Table 3A: *p* ≤ 0.0134 in all pairwise comparisons) and consistent with what has been previously described in melon (Sattar et al., 2012; Herranz et al., 2015; Sanz-Carbonell et al., 2019, 2020) and other members of the *Cucurbitaceae* family (Jagadeeswaran et al., 2012). Non-significant differences were found between stress conditions regarding the observed distribution of sRNAs sizes (Supplementary Table 3A: *p* = 0.857), nor the interaction between both factors (Supplementary Table 3A: *p* = 0.750). The effect of the stress conditions on sRNAs accumulation was evaluated by pairwise comparisons between control and treated samples. As described above, only sequences that match the conditions | log_2_*FC*| ≥ 1.25 and *p* < 0.05 were considered as significantly differentially expressed and retained for subsequent analysis (Supplementary Figure 2). A total of 35,906 unique reads fulfilled these conditions. The combinations that included cold as one of the stressors showed the most drastic alteration in sRNAs accumulation (21,592 reactive sRNAs in C-D, 20,760 in C-Sal, 23,506 in C-SD, and 21,263 in C-Sal-SD). In contrast, only 1,595 and 3,988 differentially expressed sRNAs were identified in plants treated with the combination D-Mon and D-S, respectively (Supplementary Figure 2). These results support the notion that exposition to low temperature (in any combination) is the most stressful environmental condition, resulting in the strongest alteration of the sRNA metabolism in melon (Figure 1C).

### Combined Stresses Induce a General Decrease of miRNA Expression

**FIGURE 2:**
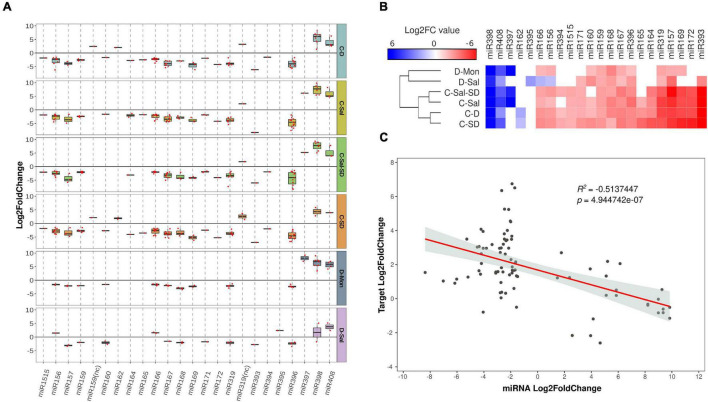
General description of stress-responsive miRNA families. **(A)** Boxplot analysis showing the general expression value observed for each miRNA family member. To determine the general sense of the expression for each miRNA family, we employed the median value of expression (represented by internal box line) estimated by boxplot analysis of all family-related sequences. The differential expression values represented in the figure correspond to the log_2_*FC* obtained using edgeR. **(B)** Heatmap of 22 miRNAs differentially expressed in melon in response to combined stress. The differential expression values represented correspond to the median of the log_2_*FC* values obtained using edgeR for each miRNA family. **(C)** A scatter plot showing the significant negative correlation (estimated by Pearson correlation coefficient) between the expression levels of 16 selected stress-responsive miRNAs with differential accumulation determined by sequencing and the accumulation of their targets in the corresponding stress conditions, estimated by RT-qPCR.

To identify melon miRNAs reactive to combined stress conditions, differentially expressed sRNAs were aligned against miRNA sequences (both mature and precursors) recovered from miRBase^4^. Only sRNAs ranging 20–22 nt and fully homologous to database sequences were considered. Two sequences homologous to mature *miR6478* but lacking a known transcript in melon with a canonical hairpin were excluded for subsequent analysis (Supplementary Figure 1). After filtering, 100 unique sequences belonging to 22 known miRNA families were identified as responsive to the combined stress conditions studied (Supplementary Table 3). In general, all family-related sequences showed a comparable trend of accumulation in response to the stress conditions analyzed (Figure 2A). A sequence variant of *miR398b* (downregulated in C-D treatment but showing a minority accumulation rate with respect to predominant family-related sequences) and the non-canonical miRNAs derived of the alternative processing of *miR319* (*miR319nc*) (Bustamante et al., 2018) and *miR159* (*miR159nc*) (Bologna et al., 2009) precursors (upregulated in cold-containing combinations and without regulatory activity described yet) showed a discordant response with the family-wise trend. In these two circumstances, the response trend of the more representative family members was considered for ulterior analysis.

The general response to stress conditions was the downregulation of miRNAs (Figure 2B). Sequences included in miRNA families *miR157, miR159, miR167, miR168, miR319*, and *miR396* showed significantly decreased accumulation in all the stress conditions analyzed. Diminished accumulation in response to stress was also observed for *miR156, miR160* (except under C-Sal-SD), *miR164, miR166, miR169* (except for D-Sal), *miR171, miR172* (except for D-Sal and D-Mon), *miR393* (except for D-Mon), *miR394*, and *miR1515*. Finally, *miR165* was downregulated in three stress conditions involving cold (C-SD, C-D, and C-Sal). Regarding miRNAs upregulated in response to stress, the *miR398* and *miR408* family-related members (except for the reads related to *miR398b* described above) showed increased accumulation in all stress conditions, whereas *miR159* was significantly overexpressed in response to C-SD and C-D, and *miR397* family was so in plants exposed to C-Sal, C-Sal-SD, and D-Mon. Sequences related to *miR156*, *miR166*, and *miR395* were specifically upregulated under D-Sal stress.

The analysis of the miRNA expression focused on each particular stress combination evidenced that cold was the most adverse environmental condition with major impact on miRNA expression in melon. A total of 20 miRNA families were reactive to C-SD and C-D and 19 to C-Sal (Figure 2B and Supplementary Table 4). While 18 miRNAs families showed differential expression under the combination of three stresses. A weaker response was associated with treatments with D-Sal (14 reactive miRNA families) and D-Mon (13 miRNAs with altered expression). Considering both stress condition and miRNA expression trend, except *miR156* and *miR166* (upregulated in D-Sal and downregulated in the other stress conditions), all miRNAs exhibit a homogenous response to the six combinations of adverse environmental conditions analyzed. The estimation of the relative accumulation of representative miRNA-precursors by RT-qPCR evidenced that only *miR398* and *miR408* showed consistence between the expression of MIR genes and the abundance of mature miRNAs in the totality of the stress conditions studied here (Supplementary Figure 3). For *miR396*, consistence was observed only in cold-containing stress combinations. In contrast, analyzed members of *miR156, miR157, miR167*, and *miR168* families exhibit, in general, antagonist accumulation when the accumulation of precursors and mature miRNAs was compared.

It has been recently proposed that certain melon miRNAs are predominantly reactive to diverse biotic and abiotic stress conditions, while other specifically respond to certain stressor and/or expositions time (Sanz-Carbonell et al., 2020). Based on this particular behavior, miRNAs belonging to both different groups were identified as stress-responsive miRNAs with *broad* and *narrow* response range, respectively, while a third group that exhibits a moderated reactivity in response to stress was identified as *intermediates*. According to our data, 10 miRNA families showed the higher response rate to combined stress, with significant differential expression (either up or down) in the six analyzed conditions (Supplementary Table 4). Eight of these miRNA families (*miR156, miR157, miR166, miR167, miR319, miR396, miR398*, and *miR408*) were mostly coincident with melon miRNAs families classified in the broad response category (*generalists*), while *miR159* and *miR168* were previously categorized as intermediates. In contrast, miRNAs with a lower response rate to double and triple stresses (responsive in three or less conditions) pervasively pertained to miRNAs families previously reported as showing *specific* response to stress conditions in melon.

To test the functional role of the miRNAs reactive to combined stresses, we analyzed the correlation between miRNA levels and transcripts accumulation in 16 representative miRNA-target modules (Supplementary Table 5) previously established and validated to occur in melon plants (Bustamante et al., 2018; Sanz-Carbonell et al., 2019, 2020). We focused on the miRNAs reactive to at least three different stress conditions (*miR156, miR159, miR160, miR164, miR166, miR167, miR169, miR171, miR172, miR319, miR393, miR396, miR397, miR398*, and *miR408*). As expected, a significant negative correlation (*r* = −0.514, 83 df, *p* < 0.001) was obtained when the expression values of stress-responsive miRNAs were compared with the accumulation (estimated by RT-qPCR) of their target transcripts (Figure 2C).

### The miRNA-Mediated Response to Stress Combinations Cannot Be Predicted From the Response to Single Stresses

**FIGURE 3:**
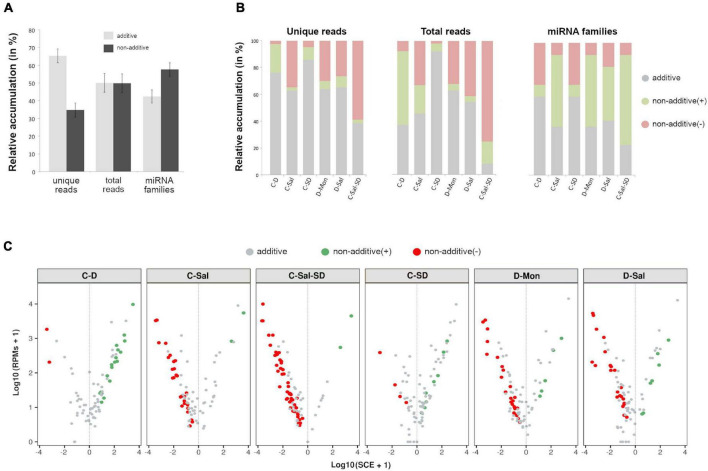
Effects of the stresses combination on the accumulation rate of stress-responsive miRNAs. **(A)** Graphic representation of the mean percentage for the six analyzed treatments of miRNA-related reads that exhibit additive (gray) or non-additive (black) response to combined stress conditions in comparison to single stresses, considering unique reads (left columns), total reads (central columns), and miRNA families (right). Bars represent the standard error between means. **(B)** The detail of the global response rate in each stress condition, considering the two (positive or negative) types of possible non-additive response to combined stresses. **(C)** A volcano plot showing significant positive (green dots) and negative (red dots) *SCE* values obtained for each miRNA-related read in response to each combined stress condition. miRNAs with non-significant deviations from the additive null model are in gray. More detailed information is provided in the Supplementary Table 6B.

To determine the dynamic of the miRNA-mediated response to multiple stress conditions, we compared the accumulation levels of stress-responsive miRNAs in plants subjected to the individual stress conditions with those of plants exposed to combined stresses. To do so, we computed *SCE* as defined in section “Materials and Methods.” Except for the combination C-Sal-SD, the additive effect was predominant in number of unique miRNA sequences in the analyzed stress combinations (65.26% of the unique reads) (Figure 3A). However, considering the entire miRNAs population (total reads), a comparable abundance of additive (50.07%) and non-additive (49.93%) instances was observed in response to combined stresses. Interestingly, when evaluating only by the miRNA family, 57.58% had at least a member showing a significant (negative or positive) *SCE* value (Figure 3A and Supplementary Table 7).

Regarding significant non-additive interactions, the stress combination predominantly exerted a negative effect in four (C-Sal, D-Sal, D-Mon, and C-Sal-SD) of the six analyzed treatments (Figure 3B). By contrast, in C-D and C-SD, *SCE* > 0 values were the most common case. Analyzing each stress combination individually, C-SD was the condition in which miRNAs show the smallest fraction of specific response to combined stresses (14.46% of unique reads, 7.77% of total reads and 40.91% of the miRNA families). In contrast, a higher differential interaction (76.47% for negative and 2.94% for positive) was observed in response to the triple combinations C-Sal-SD (61.45% of unique reads, 92.05% of total reads, and 77.27% of the miRNA families) (Figure 3B). A more general view of the additive and non-additive effects of the combined stresses onto the global population of miRNA-related reads in each analyzed stress condition is shown in the Figure 3C.

**FIGURE 4:**
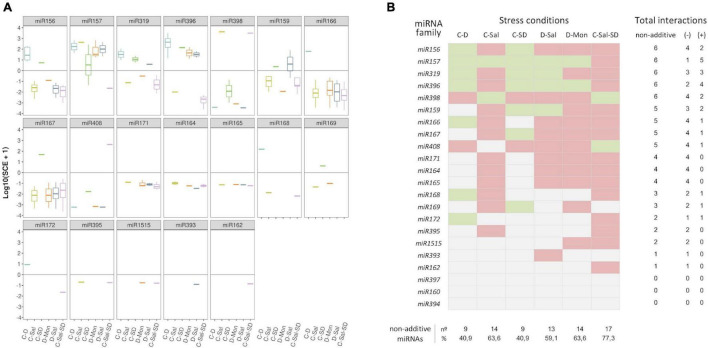
Members of each miRNA family respond in a coordinated manner to combined stresses. **(A)** Boxplot analysis showing the *SCE* values for family-miRNA-related members in each combined stress condition. To determine the general sense of the effect induced by combined stresses for each miRNA family, we employed the median of the *SCE* values obtained for the totality of the family members (represented by the internal box line). **(B)** Graphic representation of the global non-additive positive (green) or negative (red) effects associated with combined stresses estimated for each miRNA family in the six stress conditions analyzed here. The number of combined stresses that induce positive and/or negative non-additive responses in each miRNA family is detailed in the right columns. The proportion of miRNA families with non-additive effects in response to each combined stresses is detailed below.

Considering the response trend of miRNA family members, we observed that, in general, reads showed a coordinated interaction (*SCE* positive or negative) in response to the combination of stresses (Figure 4A). Consequently, a negative response was also pervasive under a global miRNA-family viewpoint. Exceptions were observed for the families *miR157* in C-SD and *miR159* in D-Sal, which contained members showing both positive and negative *SCE* values under the indicated stress combination. However, it is worth nothing that the miRNA sequences with a non-coincident trend are minority relative to the other family members (Supplementary Table 6A). Therefore, in these two specific cases, the response trend of the predominant reads was considered as representative of the family behavior for ulterior analysis (Figure 4B). The highest number (17) of miRNA families showing significant *SCE* values was observed in plants exposed to the triple combinations of stresses, followed by C-Sal and D-Mon (14) and D-Sal (13). In contrast, only nine miRNA families were identified as significantly interactive in response to C-D and C-SD, respectively.

### Different miRNA Families Act Distinctively in Response to Combined Stresses

To get further insights into the response of each miRNA family to combined stress conditions, we analyzed the rate of differential response to double and triple stresses. The 22 stress-responsive miRNA families were organized into a table of presence and absence (Supplementary Table 8) in which the values one and zero represent, respectively, whether or not a miRNA shows a significant response value (with either positive or negative effect) under a combined stress condition. Members of *miR156, miR157, miR319, miR396*, and *miR398* families showed significant positive or negative *SCE* in the six stress conditions analyzed here, while *miR159, miR166, miR167*, and *miR408* members accumulate differentially in five stresses combinations. Sequences belonging to *miR164, miR165, miR171*, and *miR393* (with positive or negative *SCE* in four conditions), *miR168* and *miR169* (in three), *miR172, miR395*, and *miR1515* (in two), and *miR162* (negative effect under C-Sal-SD) showed the lowest differential accumulation in response to the combined stress. Responsive miRNAs included in the *miR160, miR394*, and *miR397* families lacked significant interactions in any of the six analyzed stress conditions.

**FIGURE 5:**
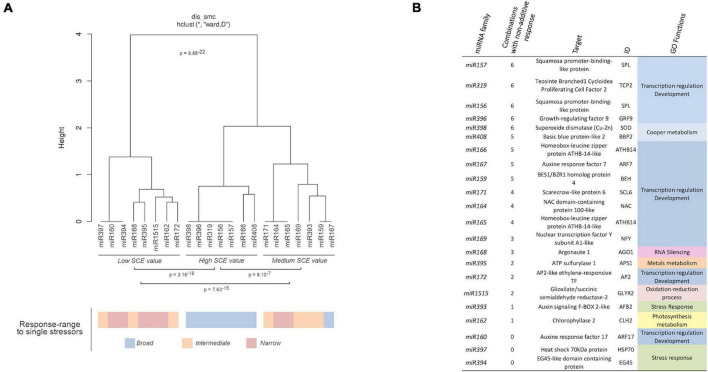
Biological functions of miRNAs with non-additive response to combined stresses. **(A)** Dendrogram showing the clustering of miRNAs families with at least a member with significant non-additive response to combined stresses in three main groups according to their *SCE* values in the analyzed stress conditions. The global statistical significance of the identified clusters (*p* = 8.88 × 10^– 22^) was estimated by Mann–Whitney–Wilcoxon test, considering the inter- and intragroup Euclidean distances. The lower panel shows the response range determined for each miRNA family in response to single stresses with both biotic and abiotic sources (using a color scale). **(B)** Description and detailed information of the targets for miRNAs with significant non-additive response to combined stresses identified in melon plants. The GO terms were estimated in a base to information of homologous transcripts in *A. thaliana*.

Correlation between miRNA responses (considering miRNA behavior and the different combined treatments) was estimated by multi-cluster analysis (MCA). MCA evidenced that the response values to combined stresses can be organized into three significantly different groups (Figure 5A). The group, including *miR156, miR157, miR166, miR319, miR396, miR398*, and *miR408*, contained the miRNA families that exclusively show significant non-additive response values (*SCE* ≠ 0 values) to combined stress conditions. In contrast, families (*miR160, miR162, miR168, miR172, miR394, miR397, miR395*, and *miR1515*) with predominantly independent responses were clustered in the second group. Families of miRNAs in which the proportion of significant (*SCE* ≠ 0 values) and non-significant (additive *SCE* values) response was comparable (*miR159, miR164, miR165, miR167, miR169, miR171*, and *miR393*) were also clustered together.

Interestingly, all the miRNAs clustered in the group showing significant non-additive expression in response to combined stresses correspond to melon miRNA families already identified as reactive to a broad range of stress (generalists) (Sanz-Carbonell et al., 2020), while miRNAs characterized by a narrow response range (specialists) are the most frequent class (five out of eight) in the group, showing mainly an additive response to double and triple stresses (Figure 5A – lower part). Finally, miRNAs identified previously as intermediates are mainly (four out of seven) included in the group where significant and non-significant responses to the combination of stressor were observed at comparable frequencies. The specialist miRNAs exhibit exclusively *SCE* < 0 response to double and triple stresses, whereas miRNAs identified as generalists showed an even distribution of significant non-additive responses (20 positive and 25 negative *SCE* values). Intermediate miRNAs, although showed a few miRNAs (five) with positive effects, were predominantly (16 miRNA families) characterized by a negative response to the combination of stresses. The relationship between miRNA trend response and stress condition was generally dependent of the specific stress/miRNA interaction, although the *miR398* and *miR408* families showed a coordinated response in all the analyzed conditions, with the exception of C-Sal. However, a positive response (*SCE* = 654.96, *p* = 0.04) was observed for *miR408* in this condition, although was considered as non-significant based on the FDR criterion (Supplementary Table 6). This specifically coordinated activity of the *miR398/miR408* tandem was particularly evident in response to C-SD and C-Sal-SD in which their response was the opposite to the general trend observed for the remaining miRNA families.

Regarding miRNA-regulated targets, it was evident that miRNAs involved in the regulation of transcription factors (TF) associated with plant development exhibit the higher rate of differential response to combined stress (Figure 5B). In contrast, miRNA families expected to modulate the expression of transcripts related (according to GO terms) to a more diverse range of biological functions (RNA silencing, metals metabolism, photosynthesis, response to stress, etc.) showed predominantly a non-significant response to stresses combination.

## Discussion

Much effort has been dedicated to elucidating the mechanisms underlying stress response in crops. Although great progress has been made in the last years, including the identification of both protein-coding and non-coding transcripts responsive to different stresses, most studies focused on deciphering the plant regulatory pathways were triggered in response to single stress conditions. Alas, no much effort has been devoted to understand the plant responses to multiple stresses acting simultaneously, a situation that is most common in the wild (Pandey et al., 2017).

Here, we have addressed this question by measuring the miRNA-mediated responses to combined stresses in melon plants exposed to five different double and one triple stressful conditions. Our strategy comprises two principal steps: first to identify the miRNA families responding to double and triple stress conditions; second, we compared the expression level of such responding miRNAs with the values obtained in melon plants exposed to the respective single stresses. This comparative analysis has allowed us to determine how the stress combinations affect the differential expression of miRNAs, disentangling stress-specific responses to general responses. This information enabled the inference of the global structure of the miRNA-mediated differential response to combined stress conditions in melon.

The computational analysis identified 22 miRNA families with significant differential expression in response to the analyzed stresses. Regarding their functional role, these reactive families mainly target melon homologous to well-described TFs [e.g., *SPOROCYTELESS, BES1/BZR1 HOMOLOG 4*, *AUXINE RESPONSE FACTORS (ARF)*, *ARABIDOPSIS THALIANA HOMEOBOX PROTEIN 14*, *TEOSINTE BRANCHED 1/CYCLOIDEA/PROLOFERATING CELL FACTOR*, *APETALA 2*, *GENERAL REGULATORY FACTOR (GR)*, and *NUCLER FACTOR Y*] This is in agreement with previous observations in other species (*A. thaliana*, rice, maize, sorghum, sunflower, etc.) in which it has been reported that, in general, miRNAs reactive to stress target predominantly TFs (Samad et al., 2017). This reinforces the emerging notion that the role played by miRNAs during the stress response is evolutionary conserved in plants (Rubio-Somoza and Weigel, 2011; Megraw et al., 2016; Sanz-Carbonell et al., 2020) and emphasizes the potential of miRNAs as targets for improving stress tolerance in crops (Tang and Chu, 2017; Chaudhary et al., 2021). The totality of these stress-responsive miRNA families was coincident with the previously described as reactive in single biotic and abiotic stress conditions in melon (Sanz-Carbonell et al., 2019, 2020). The observation that double and triple stresses do not induce the differential accumulation of any miRNA family reactive, specifically to combined stress, suggests that (at least under the conditions analyzed here), the miRNA families involved in the response to stress comprise the general structure that modulates the recovery of the plant-cell homeostasis under both single and combined adverse environmental conditions.

Considering the response rate to each stress combination, we observed a more consistent activity in certain miRNA families. Our results evidenced that melon miRNAs (*miR156, miR157, miR166, miR167, miR319, miR396, miR398*, and *miR408*) previously characterized by exhibit differential accumulation in response to a wide range of biotic and abiotic stress conditions in melon, maize, and soybean (dubbed as generalists) were differentially expressed in the six analyzed conditions, evidencing a high-response range, independently of the stresses combination. Interestingly, miRNAs families reactive to four or less conditions (*miR162, miR164, miR165, miR172, miR394, miR397, miR395*, and *miR1515*) predominantly corresponded to miRNAs characterized by exhibiting differential response to specific stresses (specialists). It has been recently suggested that generalists stress-responsive miRNAs might be involved in the modulation of the central steps in the recovery of the cell homeostasis during the exposition to adverse environmental conditions, while specialists families responding to specific stress conditions and/or exposition times had been hypothesized to be involved in the regulation of metabolic processes associated with each particular stressor (Sanz-Carbonell et al., 2019, 2020). Assuming this responsive behavior, it is expected that generalist miRNAs were the predominant class reactive to double and triple stresses. Sequences related to generalist miRNA families are characterized by mainly modulating master regulators or central hubs, predominantly TFs related with plant development (Sanz-Carbonell et al., 2020). It is well established that alteration in the expression of TF genes normally results in remarkable changes in the global gene expression during plant growth and development (Li et al., 2015). Furthermore, it has been proposed that such TFs might, for example, by co-regulatory feedback and feed-forward loops miRNA/TF, act as amplifiers of the plant response to stress (Rubio-Somoza and Weigel, 2011; Megraw et al., 2016; Samad et al., 2017). The generalist class is comprised by miRNAs previously described as reactive to different biotic and/or abiotic stress conditions in diverse plant species. Several studies support that the module *miR156*-SPLs besides exhibiting a broad response range to low temperatures in diverse plant species (Zhou and Tang, 2019) also improve tolerance to salinity, heat, and drought in *Medicago sativa* (Arshad et al., 2017a,b; Matthews et al., 2019). Moreover, the interaction between *miR396* and GRF is involved in the modulation of the response to diverse biotic (*Phytophthora nicotianae*) and abiotic (drought, salt, alkali, UV-B radiation, and osmotic unbalance) stress conditions (Gao et al., 2010; Kim et al., 2012; Casadevall et al., 2013; Chen et al., 2015). Cotton plants overexpressing *miR157* suppressed the auxin signal and showed enhanced sensitivity to heat (Ding et al., 2017). Recent studies have evidenced a critical function for *miR166* in tolerance to abiotic stresses in maize (Li et al., 2020) and Cd^++^-induced toxicity in rice (Ding et al., 2018). By means of transgenic approaches, it was established that *miR167* acts as a transcriptional regulator in response to bacterial infection (Jodder et al., 2017) and temperature-induced stress in tomato plants (Jodder et al., 2018). Multiple pieces of evidence obtained by both sRNA sequencing and transgenic approaches support the role of the members of the *miR319* family, an ancient miRNA conserved across plant species ranging from mosses to higher plants, as a key modulator of the plant-environment interrelation (at biotic and abiotic levels) in monocotyledonous and dicotyledonous species (Bustamante et al., 2018; Liu et al., 2019; Shi et al., 2019; Wu et al., 2020; Fang et al., 2021; nee Joshi et al., 2021). Finally, regarding *miR398* and *miR408* families, it has been recently proposed that these conserved miRNAs involved in the maintenance of the cooper homeostasis in plants might be also involved in the systemic signaling of the response to biotic and abiotic stresses (Burkhead et al., 2009; Sanz-Carbonell et al., 2020).

Except for *miR398* and *miR408*, we did not observe a positive relationship between the accumulation rate of certain mature miRNAs (by RNA-seq) with the estimated precursors (by RT-qPCR). This result is in coincidence with the demonstration of a frequent inconsistency between the expression of MIR genes and the abundance of mature miRNAs in plants exposed to stress conditions (Barciszewska-Pacak et al., 2015; Bustamante et al., 2018; Choi et al., 2020), which suggest the existence of an additional regulatory layer downstream transcriptional activity to control miRNA accumulation (Szweykowska-Kulinska and Jarmolowski, 2018; Manavella et al., 2019; Grabowska et al., 2020). It has been demonstrated that *CHROMATIN REMODELING FACTOR 2* (*CHR2*) acts as an ATP-dependent RNA helicase that remodels the structure of the miRNA precursors and inhibits their processing in *A. thaliana* (Wang et al., 2018). Since low temperature impacts helicases activity (Guan et al., 2013; Liu et al., 2016) and pri-RNA structures (Bustamante et al., 2018), the possibility that the significant reduction in mature miRNA accumulation observed in plants exposed to cold might be a consequence of posttranscriptional alterations of miRNA precursors that cannot be ruled out. Further studies focused on deep analysis of the transcriptional activity of MIR genes will be needed for understanding the involvement of posttranscriptional events in the regulation of the mature miRNA level in plants in response to stress.

Upon determining the melon miRNAs responsive to combined stress conditions, we attempted to analyze whether the expression of these stress-responsive miRNAs was different in comparison with that observed under each one of the stresses individually. Our conceptual premise assumes that miRNAs that did not show a significant differential (positive or negative) response to combined stresses exhibit an independent behavior to the combination of the stress conditions. The obtained results demonstrated that, in a considerable proportion of the analyzed miRNA-stress combinations (59.85%), the stress-responsive miRNAs families exhibit a differential response to the action of combined stresses. This evidences that, although the miRNAs involved in the regulation of the response to a particular stress combination are coincident with such described under individual stresses, the regulatory effects exerted on their targets are considerably different when the plant is exposed to a combination of adverse environmental conditions.

**FIGURE 6:**
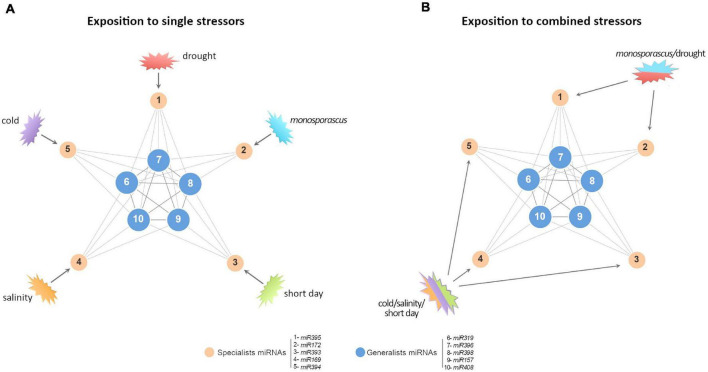
A proposed model to explain predominant non-additive response in certain miRNAs families. **(A)** Simplified graphic representation of the proposed miRNA-mediated network of response to stress in melon (Sanz-Carbonell et al., 2019, 2020). Blue nodes represent highly connected miRNAs with a broad response range to biotic and/or abiotic stress conditions (*generalists*). Orange nodes represent miRNAs reactive to specific stress conditions (*specialists*). Numbers indicate representative miRNAs for each functional group. **(B)** When the network is exposed to double (for example, *Monosporascus*/drought) or triple (cold/salinity/short day) stress conditions, it is expected that the stresses combinations should not affect specialist miRNAs (poorly connected between them), and, consequently, they exhibit additive *SCE* values (comparable to the resultant of the sum of both individual responses). In contrast, generalist miRNAs (highly interconnected) respond to stresses combination in a differential (non-additive) manner, related to each stress combination.

Considering in detail the differentially reactive miRNAs, we observed that generalist miRNAs showed the higher rate of differential accumulation (compared with the observed response to single stresses) in response to combined adverse environmental conditions, thus supporting that the biosynthesis and/or processing of such miRNA families is particularly (and differentially) susceptible to the combined exposition to two or three stress conditions. In contrast, the data obtained when miRNAs identified previously as specialists were analyzed that evidenced that the expression of this class de miRNA families is predominantly independent of the effects of the combined stresses and corresponds principally to the expression levels observed in response to each stressor individually. This functional behavior of responsive miRNAs to combined stresses is compatible with the architecture of the miRNA-mediated regulatory network of response to adverse environmental stimuli described recently in melon (Sanz-Carbonell et al., 2019, 2020). Structurally, this network is characterized by exhibiting a central core of highly connected miRNAs (generalist) and another peripheral layer comprised of miRNA families with lower connectivity (*specialists*) (Figure 6A). According to this structure, it is expected that the expression of generalist miRNAs (highly interconnected and reactive to a broad range of stress conditions) might be differentially affected (either positively or negatively) by the incidence of two or more distinct stresses (Figure 6B). In contrast, specialist miRNA families (with low connectivity and reactive to particular stress conditions) remain functionally independent to the effects of additional non-related stresses and respond mainly to the exposition to combined stress conditions in an additive (non-differential) manner (Figure 6A). The observation that the architecture of the miRNA-mediated regulatory network of response to stress in melon is able to predict the predominant reactivity rate of the miRNA response to combined stresses provides additional robustness to this inferred regulatory structure involved in the miRNA-mediated modulation of plant-environment interactions. Furthermore, the fact that structurally comparable miRNA networks of response to stress have been also proposed in rice and soybean plants exposed to diverse biotic and abiotic stress conditions (Sanz-Carbonell et al., 2020), allows to speculate about the possibility that the response pattern to combined stresses observed in melon may well be extended to another crops. In general, the transcripts of well-established TFs were the targets modulated by miRNAs with significant non-additive effects in response to combined stresses, reinforcing the key role assumed for the circuits miRNA-TF in the regulation of the stress response in plants (Rubio-Somoza and Weigel, 2011).

Regarding the trend of the global differential miRNA-mediated response to combined stresses, negative values were the most abundant. Response values lower than the expected for stress-independent effects might be initially assumed as an indicative of functional convergence in the miRNA-mediated response to combined stresses. It has been recently suggested that specific developmental events may be usually modulated by diverse miRNAs in rice (Tang and Chu, 2017). In this proposed model, miRNAs functionally converged *via* direct or indirect interaction between their targets. It is well established that osa-*miR393* regulates the auxin receptors *OsTIR1* and *OsAFB2*, both involved in the ubiquitin-mediated degradation of specific substrates during auxin signaling (Bian et al., 2012; Li et al., 2016). Furthermore, osa-*miR160* and osa-*miR167* modulate the expression of at least three *ARF* transcripts (*OsARF8*, *OsARF16*, and *OsARF18*) (Yang et al., 2006; Li et al., 2014; Huang et al., 2016). Interestingly, cmel-*miR393* and cmel-*miR167* exhibit a predominant negative differential response to the combined stresses analyzed here. Furthermore, it is expected that, according to the role of genetic redundancy in robustness (Fares, 2015), the role played by a determined cellular component (a stress-responsive miRNA in this case) may be guaranteed by another with total or partial functional overlap.

Altogether, our results support that the miRNA-mediated response to combined stress exhibits a predominant non-additive effect (indicative of that stress-responsive miRNAs might act in an interdependent and coordinated manner) mainly characterized by *SCE* < 0 values (assumed as indicators of functional convergence). Additionally, this response is a global phenomenon indistinctly triggered by diverse combination of abiotic and biotic stressors. Under a functional viewpoint, this evidence may suggest the existence of a common stress-responsive *core* (composed by non-additive miRNAs with *SCE* < 0 values) involved (by modulating pivotal TFs) in the recovering of the plant-cell homeostasis under distinct environmental pressures. On the other hand, non-additive miRNAs might be part of a more specific regulatory response to each particular stress condition. This viewing is consistent with an anticipated notion that plants may use the miRNA-mediated regulation as a pivotal mechanism to mediate the response to both simple and combined stresses (Zhang, 2015; Samad et al., 2017; Zhu et al., 2019; Zhou et al., 2020).

Finally, the confirmation that the previously described as generalist miRNAs are also the predominant components of the global miRNA-mediated response to combined stress conditions highlights the possibility that this class de miRNAs may emerge as a valuable breeding target for improving, in the near future, crop tolerance to the multiple adverse environmental conditions associated with climate change.

## Data Availability Statement

The datasets presented in this study can be found in online repositories. The names of the repository/repositories and accession number(s) can be found below: https://www.ncbi.nlm.nih.gov/, PRJNA741881.

## Author Contributions

PV-B performed and designed computational analysis, prepared figures, and discussed the results. JM-M analyzed the results, prepared figures, and contributed to wrote the manuscript. M-CM conceived and performed RT-qPCR analyses and discussed the results. AH-A performed RT-qPCR analysis. JC-S performed computational analysis. BP provided the *Monosporascus* isolate and contributed to the design of the stress treatments. AM provided melon seeds and contributed to the design of the stress treatments. SE conceived and performed the estimation of the *SCE* values and revised the manuscript. GG conceived and designed the experiments, analyzed the results, and drafted the manuscript. All the authors read and approved the final manuscript.

## Conflict of Interest

The authors declare that the research was conducted in the absence of any commercial or financial relationships that could be construed as a potential conflict of interest.

## Publisher’s Note

All claims expressed in this article are solely those of the authors and do not necessarily represent those of their affiliated organizations, or those of the publisher, the editors and the reviewers. Any product that may be evaluated in this article, or claim that may be made by its manufacturer, is not guaranteed or endorsed by the publisher.

## Footnotes

1
https://en.novogene.com

2
https://github.com/cmap/morpheus.R

3
http://rfam.xfam.org

4
http://www.mirbase.org/
