# Supplementary material for: Combined Stress Conditions in Melon Induce Non-additive Effects in the Core miRNA Regulatory Network
Source: Front Plant Sci. 2021 Nov 25;12:769093. doi: 10.3389/fpls.2021.769093 (PMC8656716; doi:10.3389/fpls.2021.769093)
Supplement: Supplementary file 1 [file Data_Sheet_1.zip › Supplementary Figures 1-3.PDF]

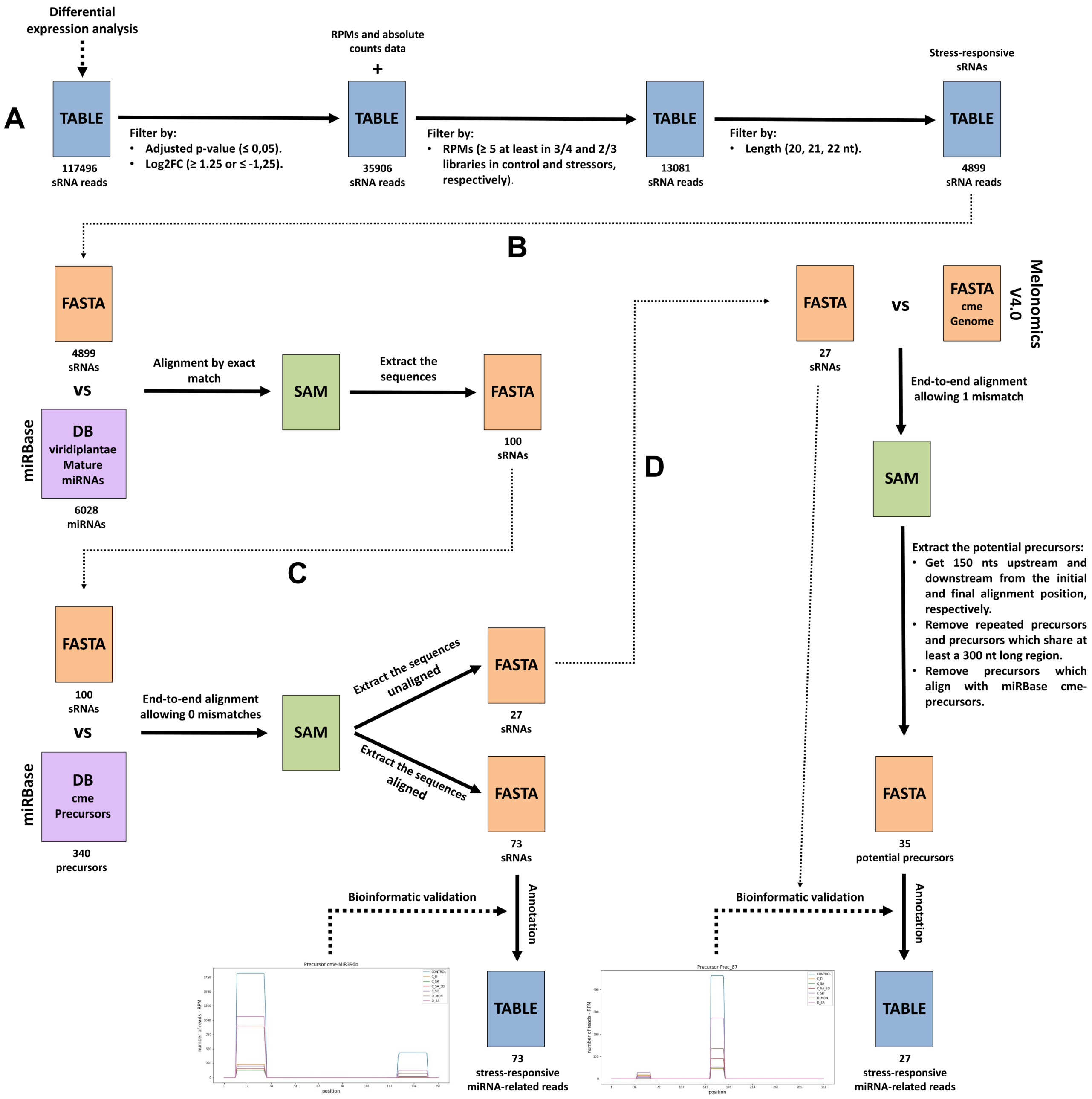

Figure S1. Pipeline for miRNA detection. A sRNA reads data coming from the differential expression analysis were filtered by adjusted p-value, log2FoldChange, RPMs and length to get the stress-responsive sRNA reads; **B** Stress-responsive sRNAs were aligned by exact match on viridiplantae mature miRNAs deposited in miRBase; **C** Stress-responsive sRNAs matched in the previous step were aligned on the cucumis melo precursors deposited in miRBase without allowing mismatches. The aligned sequences were bioinformatically validated and annotated as miRNA to be used in this work; **D** Stress-responsive sRNAs unaligned in the previous step were aligned on the cucumis melo genome regarding biological variability, that is, allowing 1 mismatch. Then, we looked for potential precursors which were used to bioinfomatically validate the sequences as miRNA. These sequences were annotated taking into account only the miRNA family of the viridiplantae mature miRNA on which aligned in the step B.

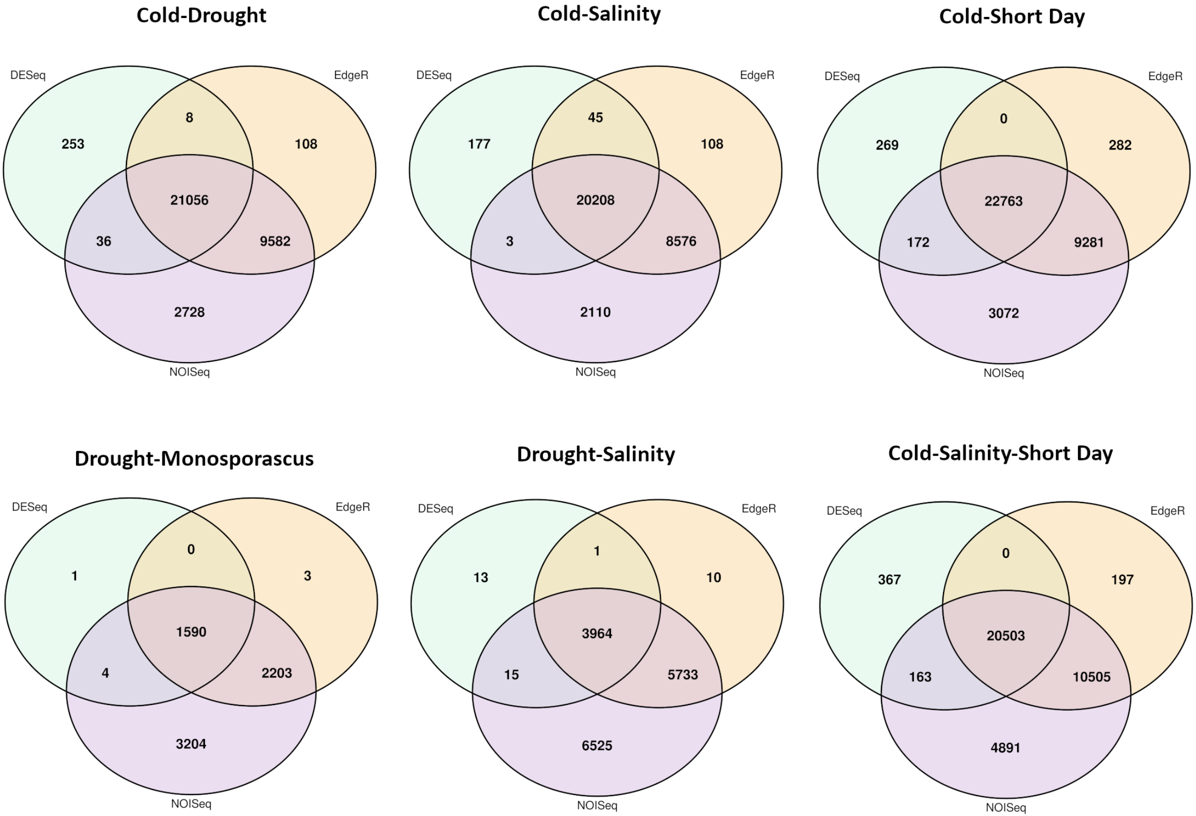

Figure S2: Analysis of stress-responsive sRNAs. Venn diagram comparing the number of the differential sRNAs -estimated by DESeq2 (green), edgeR (orange) and NOISeq (magenta)- expressed in melon in response to combined stress conditions. Only the sRNAs predicted as differential by all three analysis methods were considered as true stress-responsive.

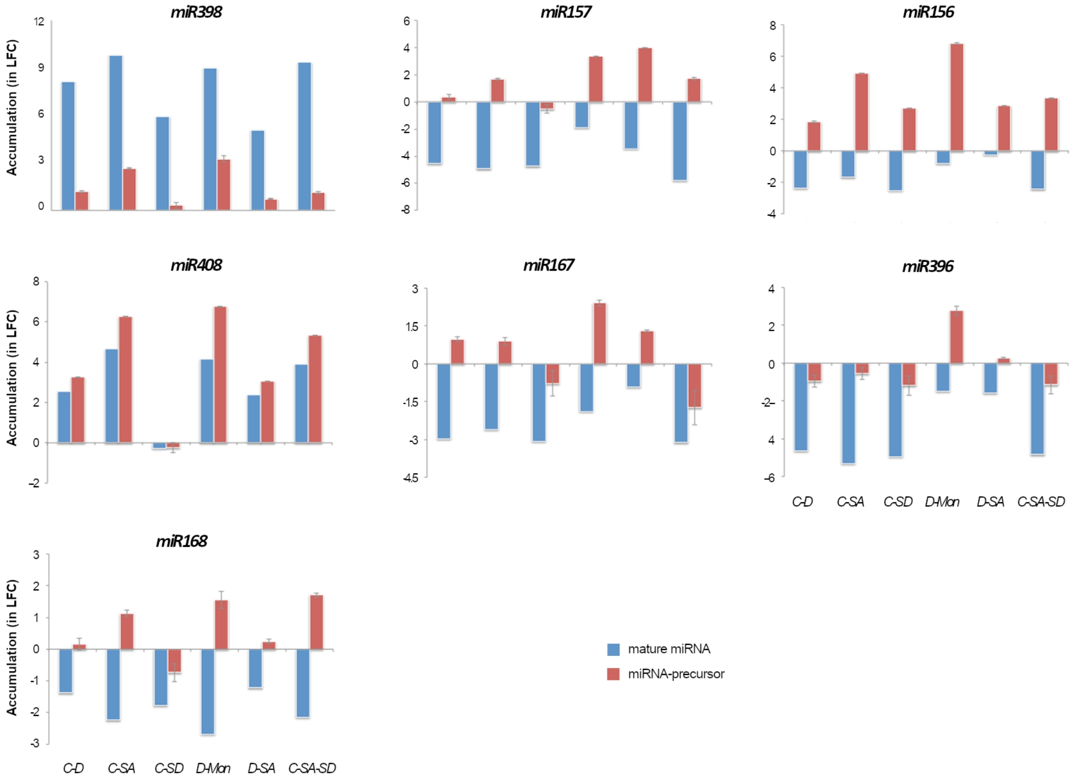

Figure S3 : Accumulation levels of mature miRNAs and miRNA-precursors in response to stress combinations are inconsistent. Graphic representation of the relative accumulation of representative mature miRNAs estimated by sequencing (blue) and their respective precursor estimated by RT-qPCR. (red). C-D: cold-drought, C-SA (cold-salinity), C-SD (cold-short day), D-Mon (drought- Monosporascus), D-SA (drought-salinity), C-SA-SD (cold-salinity-short day). Bars in the relative precursor values represent the standar error.
