# Supplementary material for: Combined Stress Conditions in Melon Induce Non-additive Effects in the Core miRNA Regulatory Network
Source: Front Plant Sci. 2021 Nov 25;12:769093. doi: 10.3389/fpls.2021.769093 (PMC8656716; doi:10.3389/fpls.2021.769093)
Supplement: Supplementary file 1 [file Data_Sheet_1.zip › Supplementary Table 1.pdf]

Table S1: Detail of the combined stress tratments used in this work.

Stress Conditions	Treatments at 11 days post emergency		sample collection
Cold Drought	Irrigated with 50 ml Hoagland's solution<br>20 °C/16 h-light --- 14 °C/8 h-darkness	Except for drought combined treatments plants were irrigated alternatively (water and Hoagland's solution) by inundation (1500 mL / 48 hs)	11 days afetr stress treatment
Drought Salinity	Irrigated with 50 ml of LiCl (200 mM)<br>28 °C/16 h-light --- 20 °C/8 h-darkness		
Cold Salinity	Irrigated with 50 ml of LiCl (200 mM)<br>20 °C/16 h-light --- 14 °C/8 h-darkness		
Cold Short-day	Irrigated with 50 ml Hoagland's solution<br>20 °C/8 h-light --- 14 °C/16 h-darkness		
Drought Monosporascus	Irrigated with 50 ml Hoagland's solution plus *M. cannonballus* mycelium (1000 UFC)<br>28 °C/16 h-light --- 20 °C/8 h-darkness		
Cold Salinity Short-day	Irrigated with 50 ml of LiCl (200 mM)<br>20 °C/8 h-light --- 14 °C/16 h-darkness		
Control	Irrigated with 50 ml Hoagland's solution<br>28 °C/16 h-light --- 20 °C/8 h-darkness		
