# Supplementary material for: Combined Stress Conditions in Melon Induce Non-additive Effects in the Core miRNA Regulatory Network
Source: Front Plant Sci. 2021 Nov 25;12:769093. doi: 10.3389/fpls.2021.769093 (PMC8656716; doi:10.3389/fpls.2021.769093)
Supplement: Supplementary file 1 [file Data_Sheet_1.zip › Supplementary Table 2.pdf]

Table S2:

Detailed information of control and stress combined libraries of Cucumis melo by sRNA length.

Sample	sRNA length	Library size	Unique sRNAs	Absolute counts	RPMs	Percentage
**Control-2**	20	3254890	89809	207400	63719.51	6.37
	21	3254890	162375	462799	142185.76	14.22
	22	3254890	158651	481894	148052.32	14.81
	23	3254890	231972	563459	173111.53	17.31
	24	3254890	868012	1333057	409555.16	40.96
	25	3254890	84481	206281	63375.72	6.34
**Control-3**	20	3638337	97378	199305	54779.15	5.48
	21	3638337	174735	464258	127601.70	12.76
	22	3638337	169323	537064	147612.49	14.76
	23	3638337	254739	787518	216449.99	21.64
	24	3638337	931871	1443990	396881.87	39.69
	25	3638337	84280	206202	56674.79	5.67
**Control-4**	20	4120101	110825	218788	53102.58	5.31
	21	4120101	220779	598124	145172.17	14.52
	22	4120101	217821	625048	151706.96	15.17
	23	4120101	326044	596350	144741.60	14.47
	24	4120101	1180433	1847971	448525.66	44.85
	25	4120101	109378	233820	56751.04	5.68
**Control-5**	20	3099997	74339	168072	54216.83	5.42
	21	3099997	147609	441991	142577.88	14.26
	22	3099997	140915	478706	154421.44	15.44
	23	3099997	201649	685367	221086.34	22.11
	24	3099997	746109	1147989	370319.39	37.03
	25	3099997	68633	177872	57378.12	5.74
**C-D-1**	20	3535271	93672	256127	72449.04	7.24
	21	3535271	163192	510797	144485.95	14.45
	22	3535271	163994	420383	118911.11	11.89
	23	3535271	220670	420602	118973.06	11.90
	24	3535271	790983	1488819	421132.92	42.11
	25	3535271	91011	438543	124047.92	12.40
**C-D-2**	20	4476774	103973	231692	51754.23	5.18
	21	4476774	199393	571488	127656.21	12.77
	22	4476774	202429	535414	119598.17	11.96
	23	4476774	273691	470427	105081.69	10.51
	24	4476774	1058297	2178447	486610.89	48.66
	25	4476774	103661	489306	109298.79	10.93
**C-D-3**	20	3167043	83415	188210	59427.67	5.94
	21	3167043	153710	447131	141182.48	14.12
	22	3167043	151266	367009	115883.81	11.59
	23	3167043	209855	340067	107376.82	10.74
	24	3167043	803346	1508164	476205.72	47.62
	25	3167043	77164	316462	99923.49	9.99
**C-Sal-1**	20	3278670	89001	250067	76270.87	7.63
	21	3278670	143661	441926	134788.19	13.48
	22	3278670	139630	367317	112032.32	11.20
	23	3278670	185759	345986	105526.33	10.55
	24	3278670	730135	1444632	440615.25	44.06
	25	3278670	74762	428742	130767.05	13.08
**C-Sal-2**	20	3711038	96057	271341	73117.28	7.31
	21	3711038	165605	650909	175398.10	17.54
	22	3711038	162507	452190	121850.01	12.19
	23	3711038	203520	437013	117760.31	11.78
	24	3711038	765211	1569523	422933.69	42.29
	25	3711038	64200	330062	88940.61	8.89
**C-Sal-3**	20	2913493	78960	242703	83303.10	8.33
	21	2913493	126927	446263	153171.12	15.32
	22	2913493	126205	348426	119590.47	11.96
	23	2913493	158297	317634	109021.71	10.90
	24	2913493	610326	1207434	414428.32	41.44
	25	2913493	62263	351033	120485.27	12.05
**C-SD-1**	20	4349145	111312	239944	55170.38	5.52
	21	4349145	198300	525716	120878.01	12.09
	22	4349145	198651	506734	116513.48	11.65
	23	4349145	277124	465703	107079.21	10.71
	24	4349145	1006814	2085161	479441.59	47.94
	25	4349145	100915	525887	120917.33	12.09
**C-SD-2**	20	4070723	101751	230724	56678.87	5.67
	21	4070723	176838	470250	115520.02	11.55
	22	4070723	183424	475225	116742.16	11.67
	23	4070723	247979	427667	105059.22	10.51
	24	4070723	916867	1884761	463003.99	46.30
	25	4070723	101174	582096	142995.73	14.30
**C-SD-3**	20	3060927	86503	227480	74317.36	7.43
	21	3060927	146348	400929	130982.87	13.10
	22	3060927	147836	408285	133386.06	13.34
	23	3060927	192491	342739	111972.29	11.20
	24	3060927	676210	1305283	426433.89	42.64
	25	3060927	62608	376211	122907.54	12.29
**D-Mon-1**	20	3434896	72864	152880	44507.90	4.45
	21	3434896	130959	460929	134190.09	13.42
	22	3434896	144473	521031	151687.56	15.17
	23	3434896	234176	687786	200234.88	20.02
	24	3434896	883783	1436127	418099.12	41.81
	25	3434896	73586	176143	51280.45	5.13
**D-Mon-2**	20	2621444	62174	128036	48841.78	4.88
	21	2621444	111173	355345	135553.15	13.56
	22	2621444	115707	372087	141939.71	14.19
	23	2621444	189409	570932	217792.94	21.78
	24	2621444	727440	1083621	413367.98	41.34
	25	2621444	56567	111423	42504.44	4.25
**D-Mon-3**	20	3649484	77741	159240	43633.57	4.36
	21	3649484	144625	434673	119105.33	11.91
	22	3649484	161018	451040	123590.07	12.36
	23	3649484	278741	713213	195428.45	19.54
	24	3649484	1073308	1716823	470428.97	47.04
	25	3649484	90693	174495	47813.61	4.78
**D-Sal-1**	20	4253376	123992	224374	52751.98	5.28
	21	4253376	214434	551760	129722.84	12.97
	22	4253376	213687	568311	133614.10	13.36
	23	4253376	297266	572260	134542.54	13.45
	24	4253376	1298718	2106407	495231.79	49.52
	25	4253376	124654	230264	54136.76	5.41
**D-Sal-2**	20	3585226	106153	244490	68193.75	6.82
	21	3585226	171457	471700	131567.72	13.16
	22	3585226	173928	476265	132841.00	13.28
	23	3585226	243561	422040	117716.43	11.77
	24	3585226	932902	1753949	489215.74	48.92
	25	3585226	114080	216782	60465.37	6.05
**D-Sal-3**	20	3444560	104040	233128	67680.05	6.77
	21	3444560	171156	489739	142177.52	14.22
	22	3444560	167156	430935	125105.96	12.51
	23	3444560	228695	393395	114207.62	11.42
	24	3444560	902364	1696530	492524.44	49.25
	25	3444560	105199	200833	58304.40	5.83
**C-Sal-SD-1**	20	4138669	101646	216941	52418.06	5.24
	21	4138669	192739	557918	134806.14	13.48
	22	4138669	185482	448279	108314.77	10.83
	23	4138669	264682	435175	105148.54	10.51
	24	4138669	1110437	2255499	544981.73	54.50
	25	4138669	84308	224857	54330.75	5.43
**C-Sal-SD-2**	20	4483072	86074	220464	49176.99	4.92
	21	4483072	178532	576425	128578.13	12.86
	22	4483072	179899	462350	103132.41	10.31
	23	4483072	255651	447117	99734.51	9.97
	24	4483072	1154005	2306011	514381.88	51.44
	25	4483072	97719	470705	104996.08	10.50
**C-Sal-SD-3**	20	4333858	94571	225872	52118.00	5.21
	21	4333858	185569	592924	136812.05	13.68
	22	4333858	180488	445661	102832.40	10.28
	23	4333858	245468	430304	99288.90	9.93
	24	4333858	1107161	2254538	520215.01	52.02
	25	4333858	97867	384559	88733.64	8.87
