# Supplementary material for: Combined Stress Conditions in Melon Induce Non-additive Effects in the Core miRNA Regulatory Network
Source: Front Plant Sci. 2021 Nov 25;12:769093. doi: 10.3389/fpls.2021.769093 (PMC8656716; doi:10.3389/fpls.2021.769093)
Supplement: Supplementary file 1 [file Data_Sheet_1.zip › Supplementary Table 3-a.pdf]

**Table S3a: Statically analysis of sRNAs-reads profiles in control and stresses exposed plants.** The differences between treatment and reads-length were analyzed by the Scheirer–Ray–Hare non-parametric test (upper). Once established that only length category shown significant alterations we used Dunn's Multiple Comparison Test to analyze the difference between 24 nt length reads and the rest of the read-size categories (lower).

| Categories | Df | Sum Sq | H | *p*.value |
|---|---|---|---|---|
| Stress | 6 | 3808 | 2.603 | 0.85676 |
| Length | 5 | 144878 | 99.028 | < 1.0E-5 |
| Stress:Length | 30 | 35813 | 24.479 | 0.74993 |

| Size Comparison | Z | *p*.unadj | *p*.adj |
|---|---|---|---|
| 20 - 24 | -8,994 | 2,38E-19 | 3,57E-18 |
| 21 - 24 | -3,323 | 8,92E-04 | 1,34E-02 |
| 22 - 24 | -4,316 | 1,59E-05 | 2,38E-04 |
| 23 - 24 | -4,643 | 3,43E-06 | 5,15E-05 |
| 25 - 24 | 7,339 | 2,15E-13 | 3,23E-12 |
