# Supplementary material for: Combined Stress Conditions in Melon Induce Non-additive Effects in the Core miRNA Regulatory Network
Source: Front Plant Sci. 2021 Nov 25;12:769093. doi: 10.3389/fpls.2021.769093 (PMC8656716; doi:10.3389/fpls.2021.769093)
Supplement: Supplementary file 1 [file Data_Sheet_1.zip › Supplementary Table 3A.pdf]

Table S3:

Log2FC values and FDR adjusted p-values of stress-responsive cucumis melo miRNAs in stress combined conditions.

		Log2FC						FDR adjusted p-value					
**Family**	**Sequence**	**C-D**	**C-Sal**	**C-SD**	**D-Mon**	**D-Sal**	**C-Sal-SD**	**C-D**	**C-Sal**	**C-SD**	**D-Mon**	**D-Sal**	**C-Sal-SD**
**miR1515**	**TCATTTTTGCGTGCAATGATCC**	-1.8262	-1.9106	-1.8672	-1.5612	-0.6802	-2.0549	2.4333e-03	1.6924e-03	1.3012e-03	5.5093e-02	3.2758e-01	1.1276e-04
**miR156**	**TGACAGAAGAGAGTGAGCACT**	-3.852	-3.6551	-3.8516	-1.3276	-1.235	-4.037	5.7326e-16	8.7426e-15	4.7611e-18	5.8087e-03	2.1434e-02	3.1497e-18
	**TGCTCACTTCTCTTTCTGTCAG**	-1.7851	-2.1303	-1.8749	-0.9603	-1.1438	-2.7107	5.5427e-04	1.8686e-04	1.5049e-04	1.6838e-01	6.4678e-02	5.6477e-08
	**GCTCACTTCTCTTTCTGTCAGC**	-2.2318	-2.3383	-2.7823	-1.3058	-1.1776	-1.8136	5.8696e-05	2.3950e-05	1.7064e-05	9.4235e-02	1.9136e-01	1.5807e-03
	**TTGACAGAAGATAGAGGGCAC**	-0.7439	-0.3275	-0.645	0.2777	1.4721	-0.8092	1.0537e-01	4.4855e-01	1.2759e-01	7.2673e-01	6.2662e-03	5.4992e-02
	**TGACAGAAGAGAGTGAGCAC**	-1.2787	-0.912	-1.398	-0.9811	-0.6568	-1.5966	8.4494e-03	3.1382e-02	1.6810e-03	9.3765e-02	2.6485e-01	1.4953e-03
	**TTGACAGAAGAGAGTGAGCAC**	-2.3495	-1.645	-2.5472	-0.8108	-0.2241	-2.4068	2.1497e-06	1.0136e-04	1.7616e-08	2.0214e-01	7.4925e-01	1.8429e-05
	**TGACAGAAGATAGAGAGCAC**	-6.2138	-4.4624	-4.0245	-1.6462	-1.0163	-3.3916	7.6816e-10	6.7234e-08	2.1738e-08	5.7382e-02	1.2845e-01	2.3747e-07
	**TTGACAGAAGATAGAGAGCAC**	-3.2796	-3.2847	-3.0934	-1.8759	-1.0704	-2.5633	2.4183e-08	2.8743e-11	4.7029e-08	7.0232e-04	9.1677e-02	2.9132e-06
	**TGACAGAAGAGAGTGAGCACA**	-1.5586	-1.844	-1.5482	-1.2227	-1.1955	-1.8364	4.6489e-04	1.6199e-05	1.0970e-03	2.2795e-02	2.9432e-02	1.0031e-03
**miR157**	**GCTCTCTATGCTTCTGTCATC**	-4.5129	-4.9225	-4.78	-1.9084	-3.4278	-5.7726	9.0220e-14	4.5197e-16	7.0493e-17	1.3931e-03	4.7131e-08	7.3458e-19
	**GCTCTCTATACTTCTGTCACC**	-1.122	-1.6962	-1.5651	0.3775	-0.3078	-2.4942	1.7788e-02	2.0262e-04	5.3437e-04	6.2826e-01	6.9246e-01	1.4036e-07
	**GCTCTCTATGCTTCTGTCATCA**	-3.1474	-3.6727	-3.8442	-2.2688	-2.8072	-4.8361	6.5879e-08	6.5059e-10	1.1643e-11	1.5751e-04	1.9114e-05	4.4540e-15
**miR159**	**TTTGGATTGAAGGGAGCTCTT**	-2.153	-2.2499	-2.4728	-0.8663	0.2274	-2.2786	6.6336e-05	3.6571e-06	1.4189e-07	2.0174e-01	7.6088e-01	8.2052e-07
	**CTTGGATTGAAGGGAGCTCT**	-0.8226	-0.9327	-1.5558	-1.0634	0.2216	-0.5983	8.7480e-02	8.2433e-02	7.3806e-04	1.7242e-01	7.7381e-01	2.1470e-01
	**TTTGGATTGAAGGGAGCTCCT**	-2.9147	-2.3173	-3.3123	-0.3582	-1.1474	-2.3209	8.1638e-05	1.5891e-03	6.8199e-05	7.7770e-01	1.7043e-01	5.5147e-04
	**TTTGGATTGAAGGGAGCTCTG**	-1.4621	-2.4859	-2.6794	-2.0315	0.2642	-1.7627	6.2916e-02	7.1098e-04	1.4787e-04	1.9548e-02	8.6141e-01	1.4646e-02
	**GAGCTCCTTGAAGTCCAATAG**	0.1227	-0.235	0.114	-1.4935	-1.9774	-1.8173	8.5483e-01	7.0936e-01	8.5846e-01	1.0707e-01	8.8960e-03	3.0638e-03
	**TTTGGATTGAAGGGAGCTCTC**	-2.6205	-2.724	-2.9943	-0.6241	0.3101	-2.0466	4.1190e-05	3.1332e-06	1.0684e-06	5.4728e-01	7.1944e-01	3.1048e-04
**miR159(nc)**	**AGCTGCTAAGCTATGGATCCC**	2.4394	0.9465	2.1398	NA	NA	0.1883	2.5369e-04	1.3140e-01	7.4427e-05	NA	NA	8.1600e-01
**miR160**	**TGCCTGGCTCCCTGTATGCC**	-0.1131	0.2115	-0.8248	-1.1101	-1.4337	-0.9296	8.7258e-01	7.7901e-01	6.9342e-02	1.4991e-01	2.9815e-02	8.8623e-02
	**TGCCTGGCTCCCTGTATGCCA**	-1.6692	-1.6329	-2.694	-1.5736	-2.669	-1.0826	2.5241e-03	2.3227e-02	9.7277e-08	1.2886e-02	4.5156e-04	5.2117e-02
**miR162**	**TCGATAAGCCTCTGCATCCAG**	0.9192	0.7431	1.5199	-0.5593	0.2844	0.6013	8.6547e-02	2.5583e-01	1.0492e-03	6.4663e-01	7.4310e-01	3.3250e-01
	**TTGATAAACCTCTGCATCCAG**	2.0154	1.2086	2.2584	NA	NA	0.748	5.8872e-04	9.4579e-02	1.7674e-05	NA	NA	2.5427e-01
**miR164**	**TGGAGAAGCAGGGCACGTGCT**	-2.7194	-2.7283	-4.054	-1.027	-1.0959	-3.0971	1.8736e-06	7.2653e-07	1.7176e-11	1.8939e-01	1.4688e-01	2.6696e-08
	**TGGAGAAGCAGGGCACGTGCA**	-0.0797	-1.373	-1.066	-0.976	-0.1509	-0.738	8.7778e-01	5.9796e-03	1.3742e-02	1.4979e-01	8.4233e-01	1.3097e-01
**miR165**	**TCGGACCAGGCTTCATCCCCC**	-2.5046	-1.8148	-3.5452	-1.4919	-0.1033	-1.0848	1.2591e-05	7.7396e-04	7.9918e-11	1.7601e-02	8.9704e-01	1.7679e-02
**miR166**	**TCTCGGACCAGGCTTCATTCT**	-1.0923	-2.2965	-1.8234	-1.338	0.3396	-0.4957	5.1378e-02	7.9540e-05	1.0250e-03	1.2990e-01	6.6802e-01	4.0374e-01
	**TCGGACCAGGCTTCATTCCT**	-0.9008	-1.6627	-1.4289	-1.0861	1.3503	-0.5577	1.5987e-01	1.9482e-03	7.4708e-03	1.8141e-01	4.1357e-02	4.6040e-01
	**TCGGACCAGGCTTCATTCCCT**	-1.9851	-2.6027	-2.8878	-1.4276	0.5737	-0.7608	5.4438e-03	8.2747e-05	2.1889e-05	1.5702e-01	5.0725e-01	2.7078e-01
	**TCGGACCAGGCTTCATTCCCCT**	-3.0204	-4.1476	-4.5407	-1.7399	-0.6711	-1.8535	1.7889e-03	5.8295e-06	3.0927e-06	1.4645e-01	5.0659e-01	1.4493e-02
	**GGAATGTTGTCTGGCTCGAGG**	-0.711	-1.7984	-1.3272	-1.3931	-0.6927	-2.6773	2.6192e-01	2.7567e-03	3.6347e-03	2.5117e-02	3.0634e-01	3.1045e-05
	**GGAATGTTGGCTGGCTCGAGG**	2.0451	0.8018	1.2374	-1.1431	1.7732	-0.8141	8.2930e-04	1.4876e-01	2.3876e-03	3.9974e-02	1.1164e-03	2.9697e-01
	**TCGGACCAGGCTTCATTCCCG**	-2.394	-3.4202	-4.2816	-2.0328	0.8623	-1.3329	8.6259e-03	2.3242e-05	3.5414e-07	5.1726e-02	5.3454e-01	7.7105e-02
	**TCGGACCAGGCTTCATTCCTC**	-2.3385	-1.8393	-2.7422	-1.5734	-0.2352	-1.8484	1.1121e-04	2.7991e-03	5.4742e-06	6.9208e-02	7.9848e-01	9.4666e-04
	**TCTCGGACCAGGCTTCATTCC**	-1.9712	-1.9307	-2.464	-2.049	-0.468	-2.1563	3.5811e-04	6.2984e-04	4.4099e-07	7.7371e-04	5.2708e-01	8.3862e-05
	**TCGGACCAGGCTTCATTCCC**	-1.7827	-1.8583	-1.9044	-1.3379	0.5517	-1.399	1.1768e-04	7.6181e-04	7.7828e-06	2.2948e-02	4.0255e-01	2.2732e-02
	**TTGGACCAGGCTTCATTCCCC**	-2.158	-0.7143	-2.1953	-1.8471	-0.5364	-1.4471	9.9113e-04	2.6135e-01	4.7869e-04	4.4771e-02	5.0900e-01	1.2414e-02
	**TCGGACCAGGCTTCATTCCCC**	-2.4631	-1.8952	-2.6746	-1.6983	-0.1267	-2.1526	3.2691e-05	1.5336e-03	6.9229e-08	8.8493e-03	8.8197e-01	2.0980e-04
	**CCGGACCAGGCTTCATTCCCC**	-2.152	-2.0074	-1.8662	-1.5471	-0.5691	-1.7161	2.0379e-04	2.6984e-03	3.6635e-04	6.1754e-02	4.4463e-01	1.3886e-03
	**TCGGACCAGGCTTCATTCCCCC**	-3.7372	-4.0326	-4.5699	-1.6204	-0.763	-1.5784	1.3800e-04	4.8881e-07	4.5937e-07	1.3251e-01	4.3777e-01	1.9755e-02
**miR167**	**TGAAGCTGCCAGCATGATCTT**	-3.9948	-4.195	-5.231	-1.6767	-1.5229	-3.4517	5.6893e-07	8.7590e-10	2.2832e-11	6.7075e-02	6.4634e-02	1.0521e-08
	**TGAAGCTGCCAGCATGATCT**	-2.8072	-3.0585	-3.6169	-1.6885	-0.918	-2.5615	6.4784e-07	9.4814e-08	1.1900e-13	2.6319e-03	2.0075e-01	6.0377e-08
	**TGAAGCTGCCAGCATGATCTG**	-2.9493	-2.5831	-3.1153	-1.8973	-0.9082	-3.1083	2.9133e-07	2.3760e-06	4.3495e-11	7.9746e-04	1.9024e-01	3.2141e-09
	**TGAAGCTGCCAACATGATCTG**	-4.8434	-2.1491	-3.097	-1.6292	-1.4076	-3.9552	1.6305e-08	5.6475e-04	1.7500e-06	6.6807e-02	4.5607e-02	2.8436e-08
	**TGAAGCTGCCAGCATGATCTC**	-3.9038	-3.9578	-4.2328	-1.3346	-1.5715	-3.0239	1.9046e-09	2.5671e-10	1.8087e-11	6.8548e-02	3.3814e-02	1.1726e-07
	**TGAAGCTGCCAGCATGATCTGC**	-5.405	-4.4806	-3.9579	-0.7351	-1.384	-4.9963	8.0132e-11	5.2892e-09	3.1324e-09	3.6809e-01	3.9154e-02	1.3593e-11
	**TGAAGCTGCCAGCATGATCTTA**	-4.5238	-4.3513	-5.3904	-2.3152	-1.2676	-4.021	1.5744e-07	3.6167e-07	2.9180e-09	1.2204e-02	9.1246e-02	1.2195e-07
	**TGAAGCTGCCAGCATGATCTA**	-3.0073	-3.4542	-3.8585	-2.05	-1.0672	-2.2014	9.5625e-06	1.2789e-08	1.9524e-10	5.8586e-03	1.6555e-01	1.9043e-04
	**TGAAGCTGCCAGCATGATCTGA**	-1.0809	-1.8255	-1.9691	-2.1163	-0.6294	-2.0037	2.4671e-02	3.9845e-04	3.3758e-05	1.6250e-03	3.3894e-01	8.4670e-05
**miR168**	**CCCGCCTTGCATCAACTGAAT**	-1.3557	-2.2231	-1.8382	-2.6997	-1.2152	-2.1407	1.0548e-02	3.2644e-06	7.4861e-05	1.1960e-07	4.3736e-02	1.7453e-06
	**TCGCTTGGTGCAGGTCGGGA**	-2.987	-3.3383	-4.569	-3.4835	-1.7745	-4.58	4.7619e-08	2.1443e-08	3.7360e-15	1.2259e-08	3.8447e-03	5.0014e-14
	**TCGCTTGGTGCAGGTCGGGAA**	-2.8744	-2.9726	-3.7136	-2.8867	-2.3366	-4.2159	4.9515e-05	3.8430e-04	1.4972e-13	2.5719e-07	1.3415e-04	2.2668e-14
**miR169**	**TAGCCAAAGATGACTTGCCTG**	-4.2493	-4.1773	-5.1841	-1.888	-0.5337	-4.3615	1.3701e-16	5.5974e-16	2.9413e-21	2.1679e-04	4.8904e-01	2.9739e-17
	**TAGCCAAAAATGACTTGCCTG**	-6.1281	-4.3742	-6.2001	-2.1214	-1.1149	-3.6655	1.2764e-06	1.3360e-05	3.8223e-07	6.2966e-02	1.9652e-01	7.6949e-06
	**TAGCCAAAAATGACTTGCCTGC**	-3.549	-2.8145	-4.3345	-2.6415	-0.7649	-4.2364	1.7820e-10	1.1046e-07	3.8446e-15	8.7770e-06	2.8671e-01	1.1465e-13
**miR171**	**TTGAGCCGCGTCAATATCTCT**	-1.9169	-1.7839	-2.4849	-0.2226	-0.2185	-0.4404	3.5785e-04	5.0602e-04	1.3463e-06	8.3217e-01	7.8618e-01	4.1106e-01
	**TTGAGCCGTGCCAATATCACG**	-1.142	-2.003	-1.3281	-1.1313	-1.7588	-1.9372	1.3441e-02	7.0831e-05	1.5432e-03	5.4220e-02	7.7756e-03	6.0441e-05
	**TGATTGAGCCGTGCCAATATC**	-0.6744	-1.1545	-1.275	-0.4085	0.1328	-2.1555	2.3241e-01	2.0392e-02	4.9412e-03	6.8551e-01	8.8720e-01	1.0763e-04
	**TGATTGAGCCGCGCCAATATC**	-0.8831	-0.6767	-1.2926	-0.8337	0.0443	-1.6657	1.0018e-01	2.1671e-01	1.2757e-02	3.6710e-01	9.6579e-01	2.5317e-03
**miR172**	**AGAATCTTGATGATGCTGCAT**	-4.1941	-4.2106	-5.2701	-1.017	-1.307	-4.0598	6.8329e-12	1.6300e-15	4.0517e-22	1.1363e-01	4.6252e-02	1.1957e-15
**miR319**	**TTGGACTGAAGGGAGCTCCT**	-3.6438	-3.9818	-4.0014	-1.5504	-0.9991	-2.2699	2.5144e-08	1.5408e-07	7.0411e-09	9.5859e-02	2.1583e-01	8.7394e-05
	**TTGGACTGAAGGGAGCTCCCT**	-4.3479	-4.4879	-4.0489	-2.0924	-1.7572	-3.811	2.8849e-17	9.2400e-21	8.5929e-17	6.7730e-06	3.4208e-03	2.3713e-16
	**TTGGACTGAAGGGAGCTCCTTC**	-1.3121	-2.6907	-1.5993	-1.4493	-0.4707	-1.812	1.0289e-02	2.1165e-05	1.2030e-03	7.2865e-02	6.0579e-01	4.2384e-04
	**ATTGGACTGAAGGGAGCTCC**	-1.7529	-5.4063	-2.1012	-0.8505	-1.3821	-7.3658	4.2992e-04	4.4135e-12	9.9507e-06	3.7232e-01	1.6519e-02	6.2439e-17
	**TTGGACTGAAGGGAGCTCCC**	-3.904	-2.9044	-3.6051	-1.3278	-1.2388	-3.3218	8.2557e-14	3.9659e-10	1.6418e-16	1.8778e-02	3.9851e-02	2.0070e-13
	**CTTGGACTGAAGGGAGCTCCC**	-3.7944	-3.5556	-3.1729	-2.8933	-2.8112	-3.7949	6.8965e-10	1.4061e-09	9.7052e-08	1.8453e-05	3.1287e-06	9.9177e-12
	**TTGGACTGAAGGGAGCTCCCA**	-4.6119	-3.8219	-4.3783	-2.0869	-1.9601	-2.8943	1.7594e-13	6.7166e-09	8.7857e-14	2.2543e-03	1.5463e-02	5.9825e-08
**miR319(nc)**	**AGCTGCCGACTCATTCATTCA**	0.9834	0.339	1.4474	-1.6554	0.0243	0.4403	8.1619e-02	6.1870e-01	8.0349e-03	1.3399e-01	9.8347e-01	5.9685e-01
	**AACTGCCGACTCATTCACTCA**	3.2251	2.2182	3.6825	-2.0877	0.626	1.8193	6.0178e-13	2.1864e-06	5.4095e-17	5.8586e-03	4.1949e-01	1.3089e-04
**miR393**	**TCCAAAGGGATCGCATTGATC**	-6.1573	-8.3687	-7.021	-1.2476	-2.7798	-6.0161	5.1350e-17	2.7448e-18	2.2880e-20	5.0897e-02	1.3090e-04	7.3458e-19
**miR394**	**TTGGCATTCTGTCCACCTCC**	-1.5479	-1.2149	-1.9971	-1.0037	-0.6221	-1.9342	1.0491e-03	1.1989e-02	3.9761e-06	1.0409e-01	4.1387e-01	9.0982e-06
**miR395**	**TGAAGTGTTTGGGGGAACTCT**	1.0624	NA	0.2797	NA	2.4123	0.0081	6.0510e-02	NA	6.6549e-01	NA	9.0228e-04	9.9327e-01
**miR396**	**TTCCACGGCTTTCTTGAACTT**	-1.7123	-2.8785	-2.2352	-0.0042	-0.5759	-1.9547	1.0744e-03	2.1860e-07	1.1331e-06	9.9759e-01	4.3756e-01	8.7149e-05
	**TTCCACAGCTTTCTTGAACTT**	-2.7765	-3.3932	-2.8307	-0.3165	-1.1372	-2.7128	4.0333e-07	5.6264e-12	8.8076e-09	7.2981e-01	8.4746e-02	5.1051e-06
	**TTCCACAGCTTTCTTGAACGT**	-3.1472	-3.7502	-4.1921	0.0115	-1.7695	-1.8985	8.6744e-08	5.4054e-08	1.2856e-11	9.9434e-01	4.3076e-02	1.6632e-02
	**TTCCACGGCTTTCTTGAACT**	-0.2452	-1.9035	-0.542	0.5215	0.7343	-1.4626	5.7693e-01	1.8058e-05	1.8380e-01	4.6458e-01	1.7325e-01	5.2901e-04
	**TTCCACAGCTTTCTTGAACT**	-2.9863	-3.8813	-3.2083	-0.6119	-0.8679	-4.0579	1.8166e-09	2.1459e-16	2.2472e-12	3.7110e-01	1.5676e-01	8.8482e-12
	**TTCCACGGCTTTCTTGAACTG**	-0.009	-0.7445	0.1452	0.972	0.7171	-1.7301	9.8688e-01	9.8522e-02	7.6485e-01	1.4350e-01	2.6908e-01	2.1365e-04
	**TTCCACAGCTTTCTTGAACTG**	-4.5911	-5.2701	-4.9724	-1.4939	-1.5639	-4.8268	1.0828e-14	3.6357e-19	1.9899e-16	1.6994e-02	8.1544e-02	3.4566e-10
	**TTCAATAAAGCTGTGGGAAG**	-5.3477	-5.6154	-5.3188	-2.2497	-1.9467	-5.8773	1.4530e-19	6.6678e-20	1.6087e-20	1.6578e-05	8.1536e-04	1.2062e-21
	**GTTCAATAAAGCTGTGGGAAG**	-5.5162	-6.7502	-5.6532	-2.6416	-2.3832	-8.2866	4.9393e-20	1.5440e-26	5.1826e-21	4.7532e-06	1.2332e-04	5.3180e-27
	**TTCCACAGCTTTCTTGAACTA**	-3.4151	-4.5723	-4.1329	-0.7317	-1.5414	-2.4687	7.1398e-06	1.6581e-08	8.4563e-09	4.9560e-01	5.1755e-02	3.7383e-04
	**GTTCAATAAAGCTGTGGGAAA**	-5.6333	-7.5864	-6.6783	-2.5086	-2.1654	-6.8873	5.8244e-15	6.2326e-19	5.2757e-19	5.9957e-05	5.3065e-04	5.8479e-19
	**GCTCAAGAAAGCTGTGGGAAA**	-4.6424	-6.3387	-6.6292	-2.2487	-3.514	-8.2971	1.8202e-14	4.6275e-17	1.7406e-20	5.8996e-05	4.9879e-08	8.3823e-21
**miR397**	**ATTGAGTGCAGCGTTGATGT**	NA	6.1389	NA	9.262	NA	5.2056	NA	1.0201e-08	NA	2.4994e-28	NA	2.4151e-04
	**TCATTGAGTGCAGCGTTGATG**	NA	NA	NA	6.9929	NA	NA	NA	NA	NA	5.7010e-13	NA	NA
**miR398**	**TGTGTTCTCAGGTCACCCCTT**	1.4037	0.1677	0.0452	0.1155	-0.5878	1.0645	4.6626e-03	7.5415e-01	9.3825e-01	9.0795e-01	4.4506e-01	1.0856e-02
	**TGTGTTCTCAGGTCACCCCT**	4.363	5.4066	NA	6.8501	NA	5.7665	1.1326e-06	2.3065e-10	NA	7.1923e-22	NA	2.6259e-09
	**TTGTGTTCTCAGGTCACCCCT**	0.1494	-1.175	-0.9255	-1.4753	-1.7513	-0.1244	7.6716e-01	5.8362e-03	4.5963e-02	2.4979e-02	2.2380e-02	8.1203e-01
	**TGTGTTCTCAGGTCGCCCCTG**	8.2075	9.8324	5.8613	8.988	5.1103	9.3475	1.0032e-27	3.4589e-31	5.9660e-18	5.8593e-36	3.4042e-11	2.7016e-33
	**CGTGTTCTCAGGTCGCCCCTG**	7.0048	8.7175	NA	7.3014	NA	8.9154	3.6711e-11	2.2855e-14	NA	7.7982e-13	NA	1.1665e-20
	**TATGTTCTCAGGTCGCCCCTG**	6.6989	8.1874	NA	5.4759	NA	7.7787	1.3749e-10	1.1827e-12	NA	1.3305e-05	NA	4.2905e-14
	**TGTGTTCCCAGGTCGCCCCTG**	6.7407	8.758	NA	7.3189	NA	8.6593	1.8928e-10	3.6912e-16	NA	2.0609e-11	NA	1.0682e-19
	**TGTGTTCTCAGGTCACCCCTG**	4.101	5.5449	NA	3.4284	NA	5.0494	2.5484e-08	1.8052e-11	NA	6.2581e-05	NA	7.1717e-14
	**TGTGTTCTCAGGTCGCCCCCG**	5.5011	7.2087	2.8026	5.4686	2.7248	7.4522	7.5702e-10	3.2271e-21	5.7476e-03	1.5036e-15	5.1711e-02	3.4479e-25
**miR408**	**TGCACTGCCTCTTCCCTGGCT**	NA	NA	NA	6.5709	5.2674	NA	NA	NA	NA	1.1301e-06	1.3655e-04	NA
	**TGCACTGCCTCTTCCCTGGCTG**	3.0526	5.1192	NA	5.1639	2.8418	3.989	5.9703e-04	1.1101e-06	NA	8.9807e-11	2.4331e-02	1.4229e-07
	**TGCACTGCCTCTTCCCTGGC**	6.2758	8.2118	3.9461	6.9188	5.1606	7.7813	1.0927e-14	5.0914e-12	6.0624e-05	1.9897e-15	1.7091e-07	1.5596e-20
	**ATGCACTGCCTCTTCCCTGGC**	2.584	4.6642	-0.3324	4.1449	2.3831	3.9201	7.0858e-07	8.3227e-09	5.6869e-01	3.6866e-12	5.6001e-05	8.4534e-12

Note:
* Grey: The miRNA is Non-Analyzed (NA) because it didn't pass the filter prior to the differential expression analysis.
† Red: The miRNA is Non-Significant in the differential expression analysis.
