# Supplementary material for: Combined Stress Conditions in Melon Induce Non-additive Effects in the Core miRNA Regulatory Network
Source: Front Plant Sci. 2021 Nov 25;12:769093. doi: 10.3389/fpls.2021.769093 (PMC8656716; doi:10.3389/fpls.2021.769093)
Supplement: Supplementary file 1 [file Data_Sheet_1.zip › Supplementary Table 4.pdf]

Table S4:

Presence and absence of stress-responsive miRNAs to a combined stress conditions in Cucumis melo.

1: stress-responsive, 0: non stress-responsive.

Family	C-D	C-Sal	C-SD	D-Mon	D-Sal	C-Sal-SD	Total
**miR156**	1	1	1	1	1	1	6
**miR157**	1	1	1	1	1	1	6
**miR159**	1	1	1	1	1	1	6
**miR166**	1	1	1	1	1	1	6
**miR167**	1	1	1	1	1	1	6
**miR168**	1	1	1	1	1	1	6
**miR319**	1	1	1	1	1	1	6
**miR396**	1	1	1	1	1	1	6
**miR398**	1	1	1	1	1	1	6
**miR408**	1	1	1	1	1	1	6
**miR160**	1	1	1	1	1	0	5
**miR169**	1	1	1	1	0	1	5
**miR171**	1	1	1	0	1	1	5
**miR393**	1	1	1	0	1	1	5
**miR1515**	1	1	1	0	0	1	4
**miR164**	1	1	1	0	0	1	4
**miR172**	1	1	1	0	0	1	4
**miR165**	1	1	1	0	0	0	3
**miR394**	1	0	1	0	0	1	3
**miR397**	0	1	0	1	0	1	3
**miR162**	1	0	1	0	0	0	2
**miR395**	0	0	0	0	1	0	1
**Total**	20	19	20	13	14	18	104
