# Supplementary material for: Combined Stress Conditions in Melon Induce Non-additive Effects in the Core miRNA Regulatory Network
Source: Front Plant Sci. 2021 Nov 25;12:769093. doi: 10.3389/fpls.2021.769093 (PMC8656716; doi:10.3389/fpls.2021.769093)
Supplement: Supplementary file 1 [file Data_Sheet_1.zip › Supplementary Table 5.pdf]

Table S5: Detail of the expression values otained for the more representative miRNA family members (by seqencing) and their respectives RNA targets (by RT-qPCR).

target-miRNA module	stress	miRNA LFC	Target LFC
miR156-SPL9	C/D	-2,35	3,41
	C/SA	-1,64	2,12
	C/SD	-2,55	3,20
	D/SA	1,47	1,22
	D/MON	-1,88	1,67
	C/SA/SD	-2,41	2,62
miR159-BEH4	C/D	-2,15	2,97
	C/SA	-2,25	4,01
	C/SD	-2,47	3,50
	D/SA	-1,98	2,30
	D/MON	-2,03	4,56
	C/SA/SD	-2,28	3,45
miR160-ARF17	C/D	-1,67	1,69
	C/SA	-1,63	2,16
	C/SD	-2,69	1,82
	D/SA	-2,67	0,38
	D/MON	-1,57	1,34
miR164-NAC	C/D	-2,72	0,05
	C/SA	-2,73	0,95
	C/SD	-4,05	-0,79
	C/SA/SD	-3,10	1,57
miR166-ATHB14	C/D	-2,46	5,26
	C/SA	-1,90	6,75
	C/SD	-2,67	6,35
	D/SA	1,77	2,70
	D/MON	-1,70	6,51
	C/SA/SD	-2,15	5,24
miR167-ARF6	C/D	-2,95	1,59
	C/SA	-2,58	1,57
	C/SD	-3,12	1,54
	D/SA	-1,57	-0,50
	D/MON	-1,90	1,51
	C/SA/SD	-3,11	1,50
miR169-NFY	C/D	-4,25	1,44
	C/SA	-4,18	2,07
	C/SD	-5,18	1,27
	D/MON	-1,89	1,87
	C/SA/SD	-4,36	1,64
miR171-SCL6	C/D	-1,92	3,67
	C/SA	-2,00	4,52
	C/SD	-2,48	3,81
	D/SA	-1,76	1,66
	C/SA/SD	-1,94	3,49
miR172-AP2	C/D	-4,19	2,64
	C/SA	-4,21	4,08
	C/SD	-5,27	3,50
	C/SA/SD	-4,06	3,95
miR319-TCP2	C/D	-4,35	3,06
	C/SA	-4,49	3,03
	C/SD	-4,05	2,96
	D/SA	-1,76	0,60
	D/MON	-2,09	1,40
	C/SA/SD	-3,81	2,96
miR393-AFB2	C/D	-6,16	0,91
	C/SA	-8,37	1,54
	C/SD	-7,02	1,04
	D/SA	-2,78	0,35
	C/SA/SD	-6,02	1,15
miR396-GRF9	C/D	-2,78	2,71
	C/SA	-3,39	3,18
	C/SD	-2,83	2,96
	D/SA	-2,38	0,32
	D/MON	-1,49	1,30
	C/SA/SD	-2,71	2,98
miR397-HSP	C/SA	6,14	2,06
	D/MON	9,26	0,53
	C/SA/SD	5,21	2,19
miR398-CUP	C/D	8,21	-0,32
	C/SA	9,83	-0,51
	C/SD	5,86	0,51
	D/SA	5,11	1,33
	D/MON	8,99	-0,03
	C/SA/SD	9,35	-0,82
miR398-SOD	C/D	8,21	-0,38
	C/SA	9,83	-1,15
	C/SD	5,86	0,19
	D/SA	5,11	0,20
	D/MON	8,99	-0,83
	C/SA/SD	9,35	-0,61
miR408-BBL2	C/D	2,58	-2,16
	C/SA	4,66	-2,60
	C/SD	3,95	-2,17
	D/SA	2,38	1,24
	D/MON	4,14	-1,19
	C/SA/SD	3,92	1,75
