# Supplementary material for: Combined Stress Conditions in Melon Induce Non-additive Effects in the Core miRNA Regulatory Network
Source: Front Plant Sci. 2021 Nov 25;12:769093. doi: 10.3389/fpls.2021.769093 (PMC8656716; doi:10.3389/fpls.2021.769093)
Supplement: Supplementary file 1 [file Data_Sheet_1.zip › Supplementary Table 6B.pdf]

Table S6B:

Log10(SCE + 1) values of stress-responsive miRNAs in Cucumis melo.

Family	sRNA	C-D	C-Sal	C-SD	D-Mon	D-Sal	C-Sal-SD
miR1515	TCATTTTTGCGTGCAATGATCC	0.205	-0.5775	0.4546	-0.7541	-0.6381	-0.79
miR156	GCTCACTTCTCTTTCTGTCAGC	0.0829	-0.3162	-0.2046	-0.5491	-0.622	-0.6858
	TGACAGAAGAGAGTGAGCACA	0.9932	-0.9311	0.6012	-0.4497	-0.9554	-1.3785
	TGACAGAAGAGAGTGAGCACT	0.856	-0.9707	0.7265	-0.7899	-1.1356	-1.4622
	TGCTCACTTCTCTTTCTGTCAG	2.2955	-1.4007	1.5016	2.0962	1.7082	-2.5608
	TTGACAGAAGAGAGTGAGCAC	2.1891	-2.6423	1.1456	-2.1953	-2.5281	-3.0071
	TTGACAGAAGATAGAGAGCAC	1.4373	-1.7235	0.2556	-1.3264	-1.6538	-2.0545
	TTGACAGAAGATAGAGGGCAC	-0.8374	-1.4539	-0.7914	-0.9082	-0.9924	-1.6467
miR157	GCTCTCTATACTTCTGTCACC	0.9692	-1.1521	-1.3609	1.2435	-0.5613	-1.7005
	GCTCTCTATGCTTCTGTCATC	2.8414	2.6475	2.4262	2.8407	2.6665	-1.966
	GCTCTCTATGCTTCTGTCATCA	1.6437	1.3138	-0.8241	1.5289	1.3492	-1.5833
miR159	AGCTGCTAAGCTATGGATCCC	0.547	-0.4997	0.3673	0.1305	-0.0939	-0.652
	GAGCTCCTTGAAGTCCAATAG	0.6604	-0.5027	-0.9771	0.4558	-0.2673	-1.3769
	TTTGGATTGAAGGGAGCTCCT	-0.0334	-0.5602	0.2852	-0.1555	-0.6839	-0.66
	TTTGGATTGAAGGGAGCTCTC	-0.9226	-1.3389	0.6968	-1.2269	-1.1464	-1.4662
	TTTGGATTGAAGGGAGCTCTG	0.0085	-1.4011	-0.6886	-1.3266	1.911	-1.5097
	TTTGGATTGAAGGGAGCTCTT	-1.4546	-1.9506	1.3783	-1.9287	-1.5142	-2.1776
miR160	TGCCTGGCTCCCTGTATGCCA	1.4254	0.5763	-1.1352	0.7522	-1.4112	-1.5688
miR162	TCGATAAGCCTCTGCATCCAG	-0.7613	-0.7768	0.2137	-0.7026	-0.039	-0.9202
	TTGATAAACCTCTGCATCCAG	0.2356	-0.5735	0.4711	-0.2702	-0.0318	-0.7952
miR164	TGGAGAAGCAGGGCACGTGCA	0.6826	-0.8174	-0.4465	-0.6793	-0.677	-1.0665
	TGGAGAAGCAGGGCACGTGCT	-0.9737	-1.1474	0.4214	-1.2283	-1.462	-1.3768
miR165	TCGGACCAGGCTTCATCCCCC	-0.296	-1.1319	0.31	-1.1089	-1.1324	-1.2174
miR166	CCGGACCAGGCTTCATTCCCC	0.1345	-0.8522	0.5157	-0.8992	-0.8886	-1.0415
	GGAATGTTGGCTGGCTCGAGG	1.8061	-1.9876	-0.9263	-1.6505	-1.3736	-2.2517
	GGAATGTTGTCTGGCTCGAGG	1.7935	-2.0966	-1.056	-1.8381	-2.0101	-2.3977
	TCGGACCAGGCTTCATTCCCC	1.7759	-3.4511	2.6882	-3.4721	-3.4195	-3.6796
	TCGGACCAGGCTTCATTCCCCC	0.6952	-1.4369	1.4086	-1.2601	-1.5238	-1.3379
	TCGGACCAGGCTTCATTCCCCT	1.8536	-2.4579	2.4163	-2.0164	-2.4177	-2.3491
	TCGGACCAGGCTTCATTCCCG	-1.0515	-1.2472	0.9904	-1.639	0.8597	-0.9963
	TCGGACCAGGCTTCATTCCCT	-2.6359	-3.1931	2.3874	-3.1117	-3.1234	-3.1315
	TCGGACCAGGCTTCATTCCTC	-0.5458	-0.8649	-0.2474	-0.978	-0.9974	-1.0853
	TCTCGGACCAGGCTTCATTCC	1.8999	-2.2848	-0.5772	-2.2991	-2.1836	-2.6313
	TCTCGGACCAGGCTTCATTCT	-1.3555	-2.0793	1.1213	-1.9835	-1.9444	-2.0008
	TTGGACCAGGCTTCATTCCCC	0.3075	0.3428	0.42	-0.6439	-0.4393	-0.5037
miR167	TGAAGCTGCCAACATGATCTG	0.1039	-0.1901	0.1852	-0.4533	-0.3785	-0.598
	TGAAGCTGCCAGCATGATCTA	1.8078	-2.4246	2.1449	-1.8587	-2.4274	-2.4603
	TGAAGCTGCCAGCATGATCTC	0.4004	-1.2635	1.1037	-1.0892	-1.4278	-1.3517
	TGAAGCTGCCAGCATGATCTG	2.9393	-3.3545	3.0106	-3.2857	-3.3652	-3.6118
	TGAAGCTGCCAGCATGATCTGA	0.0871	-0.7048	0.3552	-0.9288	-0.7209	-0.8748
	TGAAGCTGCCAGCATGATCTGC	-0.0653	-0.6987	0.3939	-0.6173	-0.8166	-0.829
	TGAAGCTGCCAGCATGATCTT	1.0557	-1.7963	1.6886	-1.4518	-1.968	-1.9198
	TGAAGCTGCCAGCATGATCTTA	-0.2589	-0.7729	0.4087	-0.7222	-0.8964	-0.8683
miR168	CCCGCCTTGCATCAACTGAAT	2.1358	-1.8696	1.7239	1.1692	1.1938	-2.2298
	TCGCTTGGTGCAGGTCGGGAA	2.2302	-0.904	1.9011	1.8964	-0.9668	-2.1238
miR169	TAGCCAAAAATGACTTGCCTG	0.45	-0.8258	0.45	0.0288	-0.7344	-0.8105
	TAGCCAAAAATGACTTGCCTGC	0.715	-1.3313	0.6278	-0.9566	-1.3059	-1.4487
	TAGCCAAAGATGACTTGCCTG	0.47	-0.8588	0.5322	-1.0592	-0.8824	-1.2212
miR171	TGATTGAGCCGCGCCAATATC	-0.667	-1.112	-0.5161	-1.2123	-1.239	-1.4447
	TGATTGAGCCGTGCCAATATC	0.4273	-0.5566	-0.6952	-0.7	-0.534	-1.1871
	TTGAGCCGCGTCAATATCTCT	-0.4265	-0.8882	0.2802	-0.535	-0.9628	-0.865
	TTGAGCCGTGCCAATATCACG	0.3336	-0.995	0.2087	-1.2716	-1.3224	-1.556
miR172	AGAATCTTGATGATGCTGCAT	0.931	-0.4473	-1.311	-0.2753	-0.4512	-1.641
miR319	AACTGCCGACTCATTCACTCA	0.991	-1.1321	1.3643	-0.5028	0.3612	-1.3234
	AGCTGCCGACTCATTCATTCA	0.4746	-0.4442	0.7579	-0.2195	0.6453	-0.5187
	CTTGGACTGAAGGGAGCTCCC	0.8094	0.1356	-0.2261	0.2183	-0.1413	-0.8651
	TTGGACTGAAGGGAGCTCCCA	0.9825	0.4082	0.8014	0.7648	0.578	-0.5567
	TTGGACTGAAGGGAGCTCCCT	2.0399	1.2766	1.5234	1.0002	1.3769	-2.119
	TTGGACTGAAGGGAGCTCCTTC	0.3422	-0.073	-0.0672	-0.1475	0.5444	-0.4171
miR393	TCCAAAGGGATCGCATTGATC	0.3709	-0.2638	-0.6522	-0.3379	-0.9032	-1.1661
miR395	TGAAGTGTTTGGGGGAACTCT	0.4485	-0.6927	-0.1711	0.2736	0.6788	-0.7418
miR396	GCTCAAGAAAGCTGTGGGAAA	0.7803	-0.3349	-0.6452	0.4826	-0.5847	-1.1031
	GTTCAATAAAGCTGTGGGAAA	1.1695	-0.1022	1.0304	1.1126	0.5173	-0.9273
	GTTCAATAAAGCTGTGGGAAG	2.511	1.7002	2.1458	2.1873	2.0012	-2.3082
	TTCCACAGCTTTCTTGAACGT	0.7188	0.496	0.6126	0.6516	-0.323	-0.3943
	TTCCACAGCTTTCTTGAACTA	2.1384	1.4162	2.1265	2.1764	1.5871	-1.6342
	TTCCACAGCTTTCTTGAACTG	2.8058	-1.3804	2.6742	1.5958	1.9194	-2.8109
	TTCCACAGCTTTCTTGAACTT	3.5025	3.1677	3.1526	3.4072	3.3923	-3.6048
	TTCCACGGCTTTCTTGAACTG	-1.9932	-1.992	-1.707	-1.5926	1.8041	-2.5032
	TTCCACGGCTTTCTTGAACTT	1.0057	0.6049	0.6131	1.1164	1.2166	-1.0678
miR397	TCATTGAGTGCAGCGTTGATG	-1.1728	0.3905	0	-1.0048	-1.1728	0
miR398	CGTGTTCTCAGGTCGCCCCTG	-0.9672	1.2052	-0.6492	-0.6467	-1.0143	1.2303
	TATGTTCTCAGGTCGCCCCTG	-0.1284	1.0873	-0.5448	-0.2717	-0.5399	0.8997
	TGTGTTCCCAGGTCGCCCCTG	-0.9798	1.2463	-0.6256	-0.6607	-0.9816	1.1638
	TGTGTTCTCAGGTCACCCCTG	-0.4704	1.1877	-0.7261	-0.4834	-0.7611	1.0627
	TGTGTTCTCAGGTCACCCCTT	0.9448	-0.9462	-0.8554	0.16	-0.5903	-0.7453
	TGTGTTCTCAGGTCGCCCCCG	-1.5124	1.3931	-1.2382	-1.2769	-1.4087	1.5699
	TGTGTTCTCAGGTCGCCCCTG	-3.4215	3.6181	-2.976	-3.1014	-3.4686	3.4992
	TTGTGTTCTCAGGTCACCCCT	1.0342	-0.7886	-0.2349	-4e-04	-0.5018	-0.6007
miR408	ATGCACTGCCTCTTCCCTGGC	-3.2175	2.8169	-1.7641	-3.1514	-3.21	2.6228
	TGCACTGCCTCTTCCCTGGCT	-1.204	0.456	0	-1.0953	-1.1574	0.364
	TGCACTGCCTCTTCCCTGGCTG	-1.429	0.9566	-0.2599	-1.3372	-1.4417	0.6225

Note:
[*] Red: The SCE is Non-Significant.
[†] Black: The SCE is Significant.
