# Supplementary material for: Combined Stress Conditions in Melon Induce Non-additive Effects in the Core miRNA Regulatory Network
Source: Front Plant Sci. 2021 Nov 25;12:769093. doi: 10.3389/fpls.2021.769093 (PMC8656716; doi:10.3389/fpls.2021.769093)
Supplement: Supplementary file 1 [file Data_Sheet_1.zip › Supplementary Table 7.pdf]

Table S7: Detail of the percentage of additive and non-additive values SCE values obtained for differentially expressed miRNAs in each analyzed stress combination.

	Stress combination effects					
	unique reads		total reads		miRNA families	
	Additive	No Additive	Additive	No Additive	Additive	No Additive
C-D	75,9	24,1	37,27	62,73	59,09	40,91
C-Sal	62,65	37,35	45,73	54,28	36,36	63,64
C-SD	85,54	14,46	92,24	7,77	59,09	40,91
D-Mon	63,86	36,14	63,01	36,99	36,36	63,64
D-Sal	65,06	34,94	54,26	45,74	40,91	59,09
C-Sal-SD	38,55	61,45	7,95	92,05	22,73	77,27
Mean in all stress	65,26	34,74	50,08	49,93	42,42	57,58
