# Supplementary material for: Combined Stress Conditions in Melon Induce Non-additive Effects in the Core miRNA Regulatory Network
Source: Front Plant Sci. 2021 Nov 25;12:769093. doi: 10.3389/fpls.2021.769093 (PMC8656716; doi:10.3389/fpls.2021.769093)
Supplement: Supplementary file 1 [file Data_Sheet_1.zip › Supplementary Table 8.pdf]

Table S8:

Presence and absence of Stress Combination Effect (SCE) for stress-responsive miRNAs in Cucumis melo.

1: non-additive SCE, 0: additive SCE.

Family	C-D	C-Sal	C-SD	D-Mon	D-Sal	C-Sal-SD	Total
miR156	1	1	1	1	1	1	6
miR157	1	1	1	1	1	1	6
miR319	1	1	1	1	1	1	6
miR396	1	1	1	1	1	1	6
miR398	1	1	1	1	1	1	6
miR159	0	1	1	1	1	1	5
miR166	1	1	0	1	1	1	5
miR167	0	1	1	1	1	1	5
miR408	1	0	1	1	1	1	5
miR171	0	1	0	1	1	1	4
miR164	0	1	0	1	1	1	4
miR165	0	1	0	1	1	1	4
miR168	1	1	0	0	0	1	3
miR169	0	1	1	0	1	0	3
miR172	1	0	0	0	0	1	2
miR395	0	1	0	0	0	1	2
miR1515	0	0	0	0	1	1	2
miR393	0	0	0	1	0	0	1
miR162	0	0	0	0	0	1	1
miR397	0	0	0	0	0	0	0
miR160	0	0	0	0	0	0	0
miR394	0	0	0	0	0	0	0
Total	9	14	9	13	14	17	76
