# Supplementary material for: Combined Stress Conditions in Melon Induce Non-additive Effects in the Core miRNA Regulatory Network
Source: Front Plant Sci. 2021 Nov 25;12:769093. doi: 10.3389/fpls.2021.769093 (PMC8656716; doi:10.3389/fpls.2021.769093)
Supplement: Supplementary file 1 [file Data_Sheet_1.zip › Supplementary Table 9.pdf]

**Table S9:** Detail of the primers used to amplification of representative miRNA-precursors.

| miRNA-precursor | Forward | Reverse |
|---|---|---|
| *cme-MIR156d* | TGGATAATTTGACAGAAGAGAGTG | AATCGATGGAGAATAATTGTGAAAC |
| *cme-MIR157* | TGACAGAAGATAGAGAGCACAGATG | GTGATGACAGAAGCATAGAGAGCAC |
| *cme-MIR167d* | TGAGAGGTTGAAGCTGCCAG | AGCATAACATCATCAAGATCC |
| *cme-MIR168* | TCGCTCAGCCTACGGTTTAC | CGGTTTCCGATTCAGTTGATGCAAG |
| *cme-MIR396b* | TTCCACAGCTTTCTTGAACTGCATC | CCCACAGCTTTATTGAACCGCAAC |
| *cme-MIR398a* | CCTACATGGTCGTCCTGAGAATAC | AGTAGCTAAATTTCAAACACTTACC |
| *cme-MIR408* | GAGCAGCGGGGAACAGACAG | ATAAGGGGCAGCCAGGGAAG |
